# Pedestrian Localization with Stride-Wise Error Estimation and Compensation by Fusion of UWB and IMU Data

**DOI:** 10.3390/s23104744

**Published:** 2023-05-14

**Authors:** Fabian Hölzke, Hagen Borstell, Frank Golatowski, Christian Haubelt

**Affiliations:** 1 Institute of Applied Microelectronics and CE, University of Rostock, 18059 Rostock, Germany; frank.golatowski@uni-rostock.de (F.G.); christian.haubelt@uni-rostock.de (C.H.); 2 Thorsis Technologies GmbH, 39114 Magdeburg, Germany; hbo@thorsis.com

**Keywords:** indoor positioning, ultra-wideband, NLOS mitigation, ZUPT, sensor fusion, industrial automation

## Abstract

Indoor positioning enables mobile machines to perform tasks (semi-)automatically, such as following an operator. However, the usefulness and safety of these applications depends on the reliability of the estimated operator localization. Thus, quantifying the accuracy of positioning at runtime is critical for the application in real-world industrial contexts. In this paper, we present a method that produces an estimate of the current positioning error for each user stride. To accomplish this, we construct a virtual stride vector from Ultra-Wideband (UWB) position measurements. The virtual vectors are then compared to stride vectors from a foot-mounted Inertial Measurement Unit (IMU). Using these independent measurements, we estimate the current reliability of the UWB measurements. Positioning errors are mitigated through loosely coupled filtering of both vector types. We evaluate our method in three environments, showing that it improves positioning accuracy, especially in challenging conditions with obstructed line of sight and sparse UWB infrastructure. Additionally, we demonstrate the mitigation of simulated spoofing attacks on UWB positioning. Our findings indicate that positioning quality can be judged at runtime by comparing user strides reconstructed from UWB and IMU measurements. Our method is independent of situation- or environment-specific parameter tuning, and as such represents a promising approach for detecting both known and unknown positioning error states.

## 1. Introduction

The increasing digitalization and networking of industrial plants enables increases in productivity and worker safety, and is an ongoing research topic. As such, networked industrial devices and people exchange increasingly rich machine-readable context information, which enables collaborative human–machine interaction in manufacturing. Reliable measurement of the positions of people is one such type of contextual information, and has a variety of safety-related and productivity-enhancing applications.

However, if a machine is to collaborate with a person (e.g., to follow them), it must be able to recognize the person and their location. While this capability is possible through machine vision, i.e., object classification and identification using camera images, the behavior of such machine learning applications is hard to predict, especially in situations not included in the training dataset. Thus, an alternative method is needed to locate people and communicate their position to a machine. Here, we argue that reliable self-localization of people through wearable or hand-held interconnected devices is a key element. If a person can be reliably located, the determined position can be easily processed and fed into the industrial network. A machine can then obtain the operator’s location information independently of its own sensors and begin autonomous locomotion.

The localization of humans is subject to limitations that machines do not have; people must carry lightweight devices, such as smartphones, that allow communication with the operating network, and these have to be equipped with sensors that facilitate localization of the person. Suitable localization methods are based on the evaluation of radio signals and inertial sensors, which can usually be detected by a smartphone and/or wearable electronics. In this work, two technologies are considered and combined: Ultra-Wideband (UWB) radio and Pedestrian Dead Reckoning (PDR).

Localization by means of UWB is accomplished by processing distance measurements between a mobile transmitter and several fixed base stations (anchors). If an object is in the line of sight between the mobile transmitter and an anchor, the distance measurement is erroneous or impossible and any derived position is subject to a bias. This state is commonly called non-line of sight (NLOS). This is contrasted by the line of sight (LOS) state in normal operation. Whether an error case exists is not always clear or predictable at runtime. For example, in industrial contexts large loads are often moved that can enter the line of sight between anchor stations and a transmitter located near the ground. Similarly, the user’s body has an attenuating effect on the useful signal. The actual noise and bias introduced by body-shadowing can vary greatly depending on the situation [1].

In addition to technical error conditions, attacks on UWB measurements that artificially lengthen or shorten the measured distances have been demonstrated [2,3,4,5]. Such attacks are not completely preventable by most UWB transmission schemes, and lead to failures even with robust methods [6]. Furthermore, sub-6 GHz 5G signals have a disruptive effect on UWB measurements [7]. This is particularly challenging in industrial networks where UWB is used for localization in parallel with local 5G networks. Thus, methods for detecting, quantifying, and compensating for erroneous measurements at runtime are critical to the reliability of positioning with UWB.

In contrast to positioning using UWB, wearable inertial measurement units (IMU) allow for direct measurement of the acceleration and rotation rate of the localized person’s body in order to measure their position change. This method is called Pedestrian Dead Reckoning (PDR) [8]. Here, the user position is tracked from a known starting point with a known starting orientation. Typically, this motion tracking is based on the detection of user strides and on the measurement or estimation of stride direction and stride length. These stride vectors are added in sequence for motion tracking. The determined stride vector is subject to error which adds up over time; thus, the accuracy of localization by PDR alone decreases with each stride. Additionally, the positioning quality depends on the quality of the initial orientation and positioning.

The accuracy and reliability of indoor localization benefits from the combination of absolute and relative positioning, such as UWB and PDR, because UWB interference usually does not affect PDR and vice versa. Moreover, PDR can continue user localization in limited time periods independently of infrastructure-based methods such as UWB.

In this work, we describe a contribution to robust indoor localization of humans in UWB-enabled environments by combining IMU-derived stride vectors and position measurements obtained by UWB. The result of the presented combination is the detection, quantification, and compensation of UWB positioning error conditions at runtime. This method is independent of assumptions about environmental factors, with the exception of an assumed minimum variance of the measurements. The evaluation is based on real measurements in different environments and on real and simulated error states. The results show that our method is superior to UWB alone and to fusion with static error variances.

The rest of this article is organized as follows. The introduction is followed by an overview of related works in Section 2. Our method is described in detail in Section 3, with a particular focus on the construction of a virtual stride vector from UWB measurements in Section 3.1, the dynamic error models in Section 3.2 and the fusion framework for corrected position estimation in Section 3.3. The method is verified by experiments described in Section 4, and the results of these experiments are provided in Section 5. Lastly, a discussion of the whole work is presented in Section 6.

## 2. Related Works

For optimal filtering of a position measurement, its uncertainty must be modeled precisely. Because this uncertainty can change at runtime, it must be estimated at runtime. This problem of estimating an error or noise covariance matrix in filtering processes has been the subject of numerous publications. For example, at the time of the publication of the Kalman filter in [9], a method for estimating the process and measurement variance by analysis of past filter results and measurements for a stationary process was demonstrated [10]. A recent review in [11] generally distinguishes between methods with and without feedback. Both variants have in common that they are based on processing past state estimates. Methods with feedback estimate the covariance matrix based on past covariance estimates. In contrast, methods without feedback use a history of the filter innovation, i.e., the differences between the state prediction and measurements. Due to this processing of past estimates and measurements, delayed adjustment of the estimated covariance matrix is always to be expected when the measurement error changes rapidly. This is especially problematic when errors due to NLOS occur during position tracking, as the transition phase from LOS to NLOS is comparatively short. Moreover, malfunctions or disturbances of a UWB system may occur abruptly. In order for such a system to be operated safely, it is important for the estimate of the reliability of this system to respond quickly to fault conditions. A distinction between the detection and mitigation approaches to handling positioning error caused by NLOS is made in [12]. While methods used for NLOS detection rule out erroneous measurements, methods for NLOS mitigation quantify and compensate for the error using alternative data sources and/or stochastic methods.

In this work, we investigate an approach to error estimation that operates independently of previous filter results. Rapid increases in the measurement error are reproduced in the same filter interval using an appropriate estimate of the error covariance matrix. Thus, erroneous position measurements are detected and their influence is reduced through a loosely coupled system of combined UWB position measurements and PDR stride vectors.

In the following, an overview of related works describing indoor positioning with UWB is provided. A particular focus is on works in which NLOS detection and mitigation have been considered and on those in which PDR has been used to handle error cases at runtime.

### 2.1. NLOS Detection without Fusion

An analysis of the maximum expected variance of a UWB position measurement in LOS and NLOS and as a function of anchor placement was performed in [13]. However, the method is not suitable for error estimation at runtime, as the actual position must be known. The sample variance of a number of past measurements was observed in [14]. The authors described a method for selecting an optimal sample variance threshold to declare an NLOS state. Here, it is assumed that there are known constant variances in LOS and NLOS. According to the threshold test, one of the two variances is assumed to be the variance of the current measurement. In [15], a UWB system was presented for safe collaboration between people and machines in a manufacturing plant. NLOS was detected by observing the channel impulse response of received signals. Localization took place only if there were enough anchor stations in LOS. In [16], the authors used a simplified model to determine the distance bias of a UWB measurement. This method is limited to UWB measurements through walls of known thickness and permittivity. The influence of the UWB incidence angle on the wall is determined together with the position by solving a minimization problem. Accordingly, this method requires a map with appropriately parameterized walls, and is not suitable for compensating for NLOS caused by moving objects in the environment. In [17], UWB measurements were classified into LOS and NLOS by comparing the signal properties with a set of reference properties under LOS and NLOS. Similarity to the references was quantified on a scale from zero to one and subjected to a test with empirically determined thresholds. Measurements classified as NLOS were assigned a scaled variance for position determination. The scaling factor was determined from the previously determined similarity measures. The (scaled) reference variances were determined empirically, and were invariant at runtime. Therefore, this method is limited to detection and compensation of previously determined error scenarios with known error characteristics. A simple method for detecting NLOS was presented in [18]. The measured distance between two anchors was compared to the known actual distance via the predetermined probability distribution of the measurement error. If the deviation of the measurement from the expected value exceeds an empirically determined threshold, NLOS between the anchors is assumed. This finding was applied to mobile transmitters located in the area between the two anchors, which are themselves under NLOS. While this method can detect cases of NLOS, it is insufficient in situations where the mobile transmitters, blocking objects, and two UWB anchors are not located on a common plane.

### 2.2. Fusion under NLOS

Positioning with UWB can be augmented with IMU measurements to improve positioning quality and help NLOS detection. The following works describe approaches to this combination in which erroneous UWB measurements are discarded.

In [19,20,21], the authors used IMU data to propagate the user position estimate. In this approach, a UWB measurement is discarded if its difference to the propagated estimate exceeds a predetermined threshold. In [22], UWB measurements were discarded if the difference between the received energy of the first path and the total received energy of the channel impulse response exceeded a threshold. A more complex approach was taken in [23], with the transition between LOS and NLOS modeled as a Markov process. The state transition was calculated by comparing the likelihood of the measurement from the predetermined error distribution under LOS, then using the probabilities for the state transition. Determination of UWB positioning was performed using a particle filter. Depending on the LOS or NLOS state, particles no longer in a plausible region were deleted. In addition, UWB positioning was fused with IMU measurements using a Kalman filter. However, the authors did not describe a procedure to adjust the sensitivity of the filter with respect to erroneous UWB measurements at runtime.

In contrast to discarding disturbed measurements, the following works selected a fitting error distribution from a number of predetermined ones. Two methods for tight and loose coupling of UWB and PDR were presented in [24]. NLOS compensation was realized either by exclusion of measurements or assumption of a lumped additional error. The channel impulse response of a received UWB signal was analyzed used in [25,26] for NLOS detection. Compensation was accomplished by selecting predetermined variances in LOS or NLOS for the measurement variance of an EKF in [25] and a particle filter in [26]. Additionally, an undefined state was considered in [26] in cases where the distinction between LOS and NLOS is unclear. Another approach was taken in [27]. Here, the transitions of the NLOS/LOS states and the change in target position were modeled as a Markov process with known locomotion velocity. The probabilities of change in the LOS/NLOS state between time steps were empirically determined depending on the environment under consideration. The UWB error distributions under LOS and NLOS were predetermined as well. The determination of the actual LOS or NLOS state and target position can be achieved with a particle filter in which the corresponding probability distributions are applied to derive the most likely current state. The authors of [28] did not describe a method to detect or quantify the bias due to NLOS; rather, they presented a method using sigma-point transformation and preceding Schmidt–Kalman filtering [29] along with state constraints [30] to achieve optimized state estimation in the presence of bias.

Yet another approach for handling erroneous UWB measurements is to scale the assumed measurement variance. The measured UWB position and predictions based on previous position filtering and user step measurements were compared in [31]. A heuristic was used to convert the difference of both points into a weighting factor for the distance measurements. This heuristic uses two pre-determined parameters that depend on the scenario considered. In [32], PDR and UWB were fused using their weighted average. The weighting was based on the number of available UWB measurements. At runtime, the exact values of the selected weights were constant and based on empirical values. In [33,34], UWB measurement and PDR were processed using a Kalman filter. The UWB measurement variance was adjusted if the Mahalanobis distance between the measurement prediction and the actual measurement exceeded a threshold. The scaling factor was determined by a heuristic depending on the Mahalanobis distance and the threshold in [33], and was determined by an iterative algorithm in [34]. In these approaches, a constant variance is assumed if the distance of the measured values remains below the limit. In [35], a position hypothesis was extrapolated from the two most recent measurements and the current acceleration. This hypothesis was offset against the current position measurement to make a position correction when any separate method detected NLOS. The authors introduced a constant correction factor depending on the empirically determined NLOS error behavior in the environment. Another approach was taken in [36], where a position was first determined through a minimization problem using the current set of UWB distance measurements.

The residuals of these measurements were then used as the parameters of an exponential scaling function populated by two other predefined parameters: an estimate of the generally expected distance residual, and the estimated variance of the distance. Using this function, the variance of the distance measurements was dynamically scaled. The scaled variance was then used in a Kalman filter to fuse IMU data and UWB measurements for position-finding. Variance scaling for a Kalman filter was applied in [37] in cases where the UWB channel impulse response indicates NLOS. Scaling was carried out using a heuristic based on the predetermined minimum and maximum values of the expected variance and the number of anchor stations in LOS and NLOS, respectively.

Instead of explicit statistical modelling and tuning of heuristics, machine learning can be applied to detect UWB measurements under NLOS and to predict and mitigate the resulting error [38]. How well these models can be generalized to varying environments and situations remains an ongoing research topic. Another approach was taken in [39], where a particle filter was used to fuse PDR and sparse UWB distance measurements using a map of the walkable environment. While this approach is suitable for environments with constrained walkable paths, such as offices, it is less applicable to open environments with changing layouts, such as workshop floors.

In summary, the works above depend on previous knowledge in the form of map data or environmental variables, which are used to tune heuristics and thresholds or to analyze the behavior of the measurement history. As such, it is unknown how well these methods fare in unknown scenarios or whether they are sluggish to respond to fast increases in error. While the safest approach is to outright ignore erroneous measurements, in this paper we argue that using a dynamically determined measurement variance is more beneficial for indoor positioning use cases, e.g., to quantify the trust in a position measurement, which allows for dynamic safety zones to be established around workers in industrial environments. In the following, we describe a method for estimating increases in the UWB positioning error at runtime and for each stride of a person. The scaling method is independent of predetermined assumptions about environmental factors, with the exception of a lower bound to guard against overconfidence in a given measurement. This dynamically determined variance is used within a loosely coupled fusion framework to combine UWB data and PDR for the continuous tracking of people.

## 3. Methods

Our method is detailed in the following sections. First, the construction of the virtual stride vector from UWB measurements is described in Section 3.1. Afterwards, the construction of the stride vector using IMU data and its error model is shown in Section 3.2.1. The error models of the virtual vector length, orientation, and translation are detailed in Section 3.2.2, Section 3.2.3 and Section 3.2.4, respectively. Finally, the loosely coupled fusion approach to mitigating the UWB positioning error is detailed in Section 3.3.

**Figure 1:**
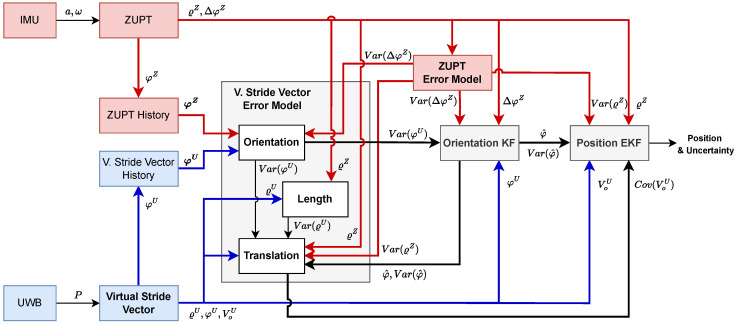
Error estimation and positioning fusion framework. The bold blocks are detailed separately in the following sections. Red elements are derived from IMU data, blue elements are derived from UWB data, and black elements are derived from combined IMU and UWB data. Here, Var(X) is the variance of a random variable *X*, Cov(X) is its covariance, *a* is the acceleration, ω is the turn rate, $\varrho^{Z}$ and $\Delta \varphi^{Z}$ are the length and orientation change of the stride vector from ZUPT, respectively, $\varphi^{Z}$ is the ZUPT vector orientation, $\varphi^{Z}$ is the set of ZUPT vector orientations, *P* is the UWB position measurement, $\varrho^{U}$ and $\varphi^{U}$ are the length and orientation of the virtual stride vector from UWB, respectively, $V_{o}^{U}$ is the vector endpoint of the virtual stride vector from UWB, and $\hat{\varphi }$ is the filtered stride orientation.

An overview of our error estimation and positioning framework is provided in Figure 1.

### 3.1. Virtual Stride Vector

The UWB measurements are converted into a virtual estimate of the user’s stride using the method of [40]. Here, the virtual stride vector ${\vec{v}}^{\;U}$ is constructed from two polar components: the estimated stride orientation $\tilde{\varphi }$, and the estimated stride length $\tilde{\varrho }$. This vector maps the relative motion of a person’s foot from stance phase to stance phase:

(1) $${\vec{v}}^{\;U}=\left(\begin{array}{c} \tilde{\varrho }\cdot cos\left(\tilde{\varphi }\right)\\ \tilde{\varrho }\cdot sin\left(\tilde{\varphi }\right) \end{array}\right)$$

The polar coordinates are obtained by statistical analysis of a set P of *n* consecutive UWB position measurements, which are modeled as realizations of the random vector P=(X,Y). Thus, a sample $P_{i}$ at time *i* is composed of realizations on the x- and y-axes of the coordinate system within which the positioning by UWB occurs. The samples are collected in the sets $X=\{X_{0},X_{1},\ldots ,X_{n-1}\}$ and $Y=\{Y_{0},Y_{1},\ldots ,Y_{n\ldots 1}\}$:

(2) $$\left(\begin{array}{c} X_{i}\\ Y_{i} \end{array}\right)=P_{i}\in P\subset R^{2}\;\mathrm{with}\;0\leq i<n$$

The set P is sampled during one whole stride of the user, which is detected by a separate stride detection scheme. The stride orientation $\tilde{\varphi }$ and stride length $\tilde{\varrho }$ are derived from an analysis of the sample covariance matrix Σ of P.

The first principal component of Σ maps the orientation and magnitude of the dominant dispersion in P, and is represented by the largest eigenvalue $\lambda_{max}=max\left(\lambda_{1},\lambda_{2}\right)$ and the corresponding eigenvector $v(\lambda_{max})$ of Σ. The dispersion along this component is mainly generated by the locomotion of the user. The eigenvalues $\lambda =(\lambda_{1},\lambda_{2})$ and the corresponding eigenvectors $v=(v_{1},v_{2})$ are found by solving the eigenvalue problem

(3) $$0=(\Sigma -\lambda I_{2})v.$$

The eigenvector $v(\lambda_{max})$ can now be used as an estimate of the direction of motion. A check of the cosine similarity against the history of UWB measurements is used to determine the mirroring of the motion vector:

(4) $$\vec{r}=\left(\begin{array}{c} r_{x}\\ r_{y} \end{array}\right)=v\left(\lambda_{max}\right)\cdot sgn\left(\left(P_{n-1}-P_{0}\right)\cdot v\left(\lambda_{max}\right)\right)$$

with sgn() as the signum function. The direction vector is translated into the desired orientation angle $\tilde{\varphi }$ of the virtual stride vector:

(5) $$\tilde{\varphi }=\mathrm{atan}2\left(r_{y},r_{x}\right)$$

The length of the virtual stride vector $\tilde{\varrho }$ is estimated using a model of the UWB measurement distribution during one user stride, as per [40]:

(6) $$\tilde{\varrho }=\sqrt{\frac{12n}{n+1}\left(\lambda_{max}-\lambda_{min}\right)}$$

Here, *n* is the UWB sample count in the measurement set P for one stride.

We continue to use the estimate of stride length from UWB data $\tilde{\varrho }$ as $\varrho^{U}$ and the estimate of stride orientation $\tilde{\varphi }$ as $\varphi^{U}$. These estimates are compared to their respective values $\varrho^{Z}$ and $\varphi^{Z}$ derived from IMU measurement.

In order to use the virtual stride vector ${\vec{v}}^{\;U}$ for position estimation in the absolute coordinate system of the localized person, its position in this reference frame must first be determined. The basis for this is the mean value of the UWB sample $\bar{P}=\frac{1}{n}\sum_{i=0}^{n-1}P_{i}$. This value determines the midpoint of the virtual stride. Thus, the absolute coordinates of the endpoint $V_{e}^{U}$ and the starting point $V_{o}^{U}$ of the virtual stride vector are provided by

(7a) $$V_{o}^{U}=\bar{P}-\frac{\varrho^{U}}{2}\frac{\vec{r}}{∥\vec{r}∥}$$

(7b) $$V_{e}^{U}=\bar{P}+\frac{\varrho^{U}}{2}\frac{\vec{r}}{∥\vec{r}∥}$$

where $\vec{r}$ denotes the direction vector from (4); thus, it holds that

(8) $$\frac{\vec{r}}{∥\vec{r}∥}=\left(\begin{array}{c} \cos(\varphi^{U})\\ \sin(\varphi^{U}). \end{array}\right)$$

### 3.2. Error Models

In general, we aim to estimate the error of measured or derived variables in the form of their variance at runtime. In certain cases, only a single estimate of the current deviation from the expected value of these quantities is available, i.e., an estimate of the mean absolute deviation (MAD) of a measured quantity *X* from its expected or mean value $\mu_{x}$: $E[|X-\mu_{x}]|$. The estimate of MAD can be translated into an estimate of variance. For a normally distributed variable $X∼N(\mu_{x},\sigma_{x})$, the following relation between the MAD and the variance holds:

(9) $$\mathrm{MAD}\left(X\right)=E[|X-\mu_{x}\left|\right]=\sigma_{x}\sqrt{\frac{2}{\pi }}$$

By rearranging the above relation, it is possible to infer the variance from the MAD:

(10) $${\sigma_{x}}^{2}=\frac{\pi }{2}\cdot {\mathrm{MAD}(X)}^{2}$$

In the following, we use this relation to derive the variance from an estimate of the MAD. We develop the error models of a stride vector measured with a foot-mounted IMU through the zero-velocity-update (ZUPT) method described in Section 3.2.1. The error models of the virtual step vector described in Section 3.1 are detailed in Section 3.2.2, Section 3.2.3 and Section 3.2.4 for its length, orientation, and translation, respectively.

#### 3.2.1. ZUPT Stride Vector

In order to generate the stride vectors for PDR, we used the zero-velocity-update (ZUPT) method with a foot-mounted IMU, as described in [41,42]. Detailed error models for ZUPT-based PDR are the subject of ongoing research, and are mostly limited to specific ZUPT methods [43,44,45]. Our PDR Error model is comparatively simple. The empirically determined expectation values of the absolute measurement error or those taken from the literature are translated into the dynamic variance estimate of the measurements using (10).

The error $\varepsilon_{\varrho^{Z}}$ of the measured stride length $\varrho^{Z}$ is scaled linearly with the measurement. The empirically determined factor $d_{\varrho^{Z}}$ scales this error:

(11) $$E[|\varepsilon_{\varrho^{Z}}\left|\right]\approx \varrho^{Z}\cdot d_{\varrho^{Z}}$$

This provides the variance estimate of the length measurement:

(12) $$\mathrm{Var}\left(\varrho^{Z}\right)=\mathrm{Var}\left(\varepsilon_{\varrho^{Z}}\right)\approx \frac{\pi }{2}{\left(\varrho^{Z}\cdot d_{\varrho^{Z}}\right)}^{2}$$

The error of the orientation change $\varepsilon_{\Delta \varphi^{Z}}$ is determined by the gyroscope drift $d_{\varphi^{Z}}$ taken from the data sheet of the sensor and the time difference relative to the previous stride:

(13) $$E[|\varepsilon_{\Delta \varphi^{Z}}\left|\right]\approx \Delta t\cdot d_{\varphi^{Z}}$$

The variance estimate of the angle measurement Δφ is obtained as follows:

(14) $$\mathrm{Var}\left(\Delta \varphi \right)=\mathrm{Var}\left(\varepsilon_{\Delta \varphi^{Z}}\right)\approx \frac{\pi }{2}{\left(\Delta t\cdot d_{\varphi^{Z}}\right)}^{2}$$

#### 3.2.2. Virtual Stride Vector—Length

The error of the length $\varrho_{i}^{U}$ of the virtual step vector ${\vec{v}}_{i}^{\;U}$ at time *i* is determined by comparing it with the corresponding stride length $\varrho_{i}^{Z}$ from ZUPT. The MAD of the actual error $\varepsilon_{\varrho_{i}^{U}}$ is estimated by the measurement $e_{\varrho_{i}^{U}}$:

(15) $$\mathrm{MAD}\left(\varepsilon_{\varrho_{i}^{U}}\right)\approx e_{\varrho_{i}^{U}}=\varrho_{i}^{U}-\varrho_{i}^{Z}$$

The squared estimate of this MAD is then converted to an estimate of the variance $\mathrm{Var}\left(\varepsilon_{\varrho_{i}^{U}}\right)=\mathrm{Var}\left(\varrho_{i}^{U}\right)$, as described in (10). However, because the reference measurement $\varrho_{i}^{Z}$ is itself subject to error (as described in (12)), $\mathrm{MAD}(\varepsilon_{\varrho_{i}^{U}})$ is estimated via an error-prone $e_{\varrho_{i}^{U}}$ and is not determined exactly:

(16) $$e_{\varrho_{i}^{U}}∼N\left(\mathrm{MAD}\left(\varepsilon_{\varrho_{i}^{U}}\right),\mathrm{Var}\left(\varepsilon_{\varrho_{i}^{Z}}\right)\right)$$

In the following, we derive a method to compensate for the noise of $e_{\varrho_{i}^{U}}$.

The distribution of the squared and normalized measurement ${\left(\frac{e_{\varrho_{i}^{U}}}{\sqrt{\mathrm{Var}(\varepsilon_{\varrho_{i}^{Z}})}}\right)}^{2}$ provided by

(17) $$\frac{e_{\varrho_{i}^{U}}}{\sqrt{\mathrm{Var}(\varepsilon_{\varrho_{i}^{Z}})}}∼N\left(\frac{\mathrm{MAD}(\varepsilon_{\varrho_{i}^{U}})}{\sqrt{\mathrm{Var}(\varepsilon_{\varrho_{i}^{Z}})}},1\right)$$

is investigated. It adheres to a non-central chi-squared distribution with degree of freedom k=1, which is because only one realization of this distribution is available at time *i*. The second parameter of this distribution is the non-centrality parameter λ, which represents the squared expected value of the underlying random variable normalized to variance 1:

(18) $$\lambda =E{\left[\frac{e_{\varrho_{i}^{U}}}{\sqrt{\mathrm{Var}(\varepsilon_{\varrho_{i}^{Z}})}}\right]}^{2}=\frac{{\mathrm{MAD}(\varepsilon_{\varrho_{i}^{U}})}^{2}}{\mathrm{Var}(\varepsilon_{\varrho_{i}^{Z}})}$$

Thus, after it is rescaled by $\mathrm{Var}(\varepsilon_{\varrho_{i}^{Z}})$, this parameter corresponds exactly to the quantity ${\mathrm{MAD}(\varepsilon_{\varrho_{i}^{U}})}^{2}$ that we seek in order to determine $\mathrm{Var}(\varrho_{i}^{U})$ through (10).

It has been shown in [46] that the following rule produces a maximum likelihood estimator (MLE) $\lambda^{*}$ of the λ parameter:

(19) $$\lambda^{*}=\max\left(\frac{{e_{\varrho_{i}^{U}}}^{2}}{\mathrm{Var}(\varepsilon_{\varrho_{i}^{Z}})}-1,0\right)$$

Thus, the following estimate of ${\mathrm{MAD}(\varepsilon_{\varrho_{i}^{U}})}^{2}$ is now available:

(20) $$\begin{array}{cc} {\mathrm{MAD}(\varepsilon_{\varrho_{i}^{U}})}^{2} & \approx \lambda^{*}\cdot \mathrm{Var}\left(\varepsilon_{\varrho_{i}^{Z}}\right)\\ \; & \approx \max\left(\frac{{e_{\varrho_{i}^{U}}}^{2}}{\mathrm{Var}(\varepsilon_{\varrho_{i}^{Z}})}-1,0\right)\cdot \mathrm{Var}\left(\varepsilon_{\varrho_{i}^{Z}}\right)\\ \; & \approx \max\left({e_{\varrho_{i}^{U}}}^{2}-\mathrm{Var}\left(\varepsilon_{\varrho_{i}^{Z}}\right),0\right) \end{array}$$

Lastly, the corrected squared estimate of the MAD is converted to an estimate of the variance $\mathrm{Var}\left(\varepsilon_{\varrho_{i}^{U}}\right)=\mathrm{Var}\left(\varrho_{i}^{U}\right)$, as described in (10):

(21) $$\begin{array}{cc} \mathrm{Var}\left(\varrho_{i}^{U}\right)=\mathrm{Var}\left(\varepsilon_{\varrho_{i}^{U}}\right) & \approx \max\left[\frac{\pi }{2}\left(e_{\varrho_{i}^{U}}^{2}-\mathrm{Var}\left(\varepsilon_{\varrho_{i}^{Z}}\right)\right),\bar{\mathrm{Var}(\varepsilon_{\varrho^{U}})}\right]\\ \; & \approx \max\left[\frac{\pi }{2}\left({\left(\varrho_{i}^{U}-\varrho_{i}^{Z}\right)}^{2}-\mathrm{Var}\left(\varepsilon_{\varrho_{i}^{Z}}\right)\right),\bar{\mathrm{Var}(\varepsilon_{\varrho^{U}})}\right] \end{array}$$

Here, the average expected error $\bar{\mathrm{Var}(\varepsilon_{\varrho^{U}})}$ serves as the minimum value of the estimate.

#### 3.2.3. Virtual Stride Vector—Orientation

The error of the orientation estimation from UWB data is expressed as the variance of the orientation $\varphi^{U}$ of the virtual UWB vector ${\vec{v}}^{\;U}$. The measurement of the orientation change $\Delta \varphi^{Z}$ from ZUPT serves as the reference value here.

The orientation measurement is scattered around the actual orientation $\varphi_{i}^{0}$ at time *i*:

(22) $$\varphi_{i}^{U}=\varphi_{i}^{0}+\varepsilon_{\varphi_{i}^{U}}$$

The error of the orientation measurement $\varepsilon_{\varphi_{i}^{U}}$ is normally distributed and changes with each stride at time *i*:

(23) $$\varepsilon_{\varphi_{i}^{U}}∼N\left(0,{\sigma_{\varphi_{i}^{U}}}^{2}\right)$$

It is assumed that an orientation error can persist over several strides and that this persistence becomes less likely over time. This consideration results in the approach presented here for estimating the error of the orientation $\varphi_{i}^{U}$ by estimating the error component $\varepsilon_{\varphi_{i}^{U}}$ of a stride at time *i*. This is based on the history of orientations taken from ZUPT $\varphi_{i}^{Z}$ at that time, which are compared with the history of orientations $\varphi_{i}^{U}$ from UWB. Thus, it holds that

(24) $$\varphi_{i}^{U/Z}=\left\{\varphi_{i-k}^{U/Z}|0\leq k<n\right\}$$

with history length *n* and 1<n<i.

The ZUPT error component $\varepsilon_{\varphi_{i}^{Z}}$ is scattered around an unknown offset $b_{\varphi_{i}^{Z}}$:

(25) $$\varepsilon_{\varphi_{i}^{Z}}∼N\left(b_{\varphi_{i-1}^{Z}},{\sigma_{\varphi_{i}^{Z}}}^{2}\right)$$

This offset is composed of the random initial orientation of the relative orientation tracking by ZUPT $b_{\varphi_{0}^{Z}}$ and the sum of the measurement errors of the following direction change $\varepsilon_{\Delta \varphi^{Z}}$ (13):

(26) $$b_{\varphi_{i-1}^{Z}}=b_{\varphi_{0}^{Z}}+\sum_{m=1}^{i-1}\varepsilon_{\Delta \varphi_{m}^{Z}}$$

Now, the first step is to estimate this offset, including the realization of the current measurement error $\varepsilon_{\Delta \varphi_{i}^{Z}}$, i.e., $b_{\varphi_{i}^{Z}}=b_{\varphi_{i-1}^{Z}}+\varepsilon_{\Delta \varphi_{i}^{Z}}$. Then, the last orientation from ZUPT $\varphi_{i}^{Z}$ rotated by $-b_{\varphi_{i}^{Z}}$ is treated as an estimate of the actual orientation $\varphi_{i}^{0}$:

(27) $$\varphi_{i}^{0}\approx \varphi_{i}^{Z}-b_{\varphi_{i}^{Z}}$$

The difference between the estimate of $\varphi_{i}^{0}$ and the measurement $\varphi_{i}^{U}$ is treated as the realized orientation error $e_{\varphi_{i}^{U}}$ (see (22)); finally $\mathrm{Var}\left(\varphi_{i}^{U}\right)=\mathrm{Var}\left(\varepsilon_{\varphi_{i}^{U}}\right)$ is derived from it using (10)

(28) $$\mathrm{Var}\left(\varphi_{i}^{U}\right)=\frac{\pi }{2}\cdot e_{\varphi_{i}^{U}}^{2}\;\mathrm{with}\;e_{\varphi_{i}^{U}}=\varphi_{i}^{U}-\varphi_{i}^{0}$$

A fundamental assumption in this approach is that the orientation errors of the virtual vectors cancel on average, while the orientation error from ZUPT is zero in the first stride (except for the random starting orientation $b_{\varphi_{0}^{Z}}$) and increases with each subsequent stride:

(29a) $$\varphi_{0}^{Z}∼N\left(\varphi_{0}^{0}+b_{\varphi_{0}^{Z}},0\right)$$

(29b) $$\varphi_{i}^{Z}∼N\left(\varphi_{i}^{0}+b_{\varphi_{0}^{Z}},\sum_{m=1}^{i}{\sigma_{\Delta \varphi_{m}^{Z}}}^{2}\right)\;\mathrm{with}\;i>0$$

Because the measurements from ZUPT are relative, this error propagation works both ways; if the unknown offset $b_{\varphi_{i}^{Z}}$ of the current stride from ZUPT $\varphi_{i}^{Z}$ is to be calibrated using the true orientation $\varphi_{i}^{0}$, the past strides $\varphi_{i-k}^{Z}$ are considered to be increasingly inaccurate references.

Thus, the error of the last orientation $b_{\varphi_{i}^{Z}}$ remains constant, while the measurements of the change of direction (The true direction difference $\Delta \varphi_{m}^{0}$ plus the measurement error $\varepsilon_{\Delta \varphi_{m}^{Z}}$) are gradually subtracted:

(30) $$\begin{array}{cc} \varphi_{i-k}^{Z} & =\varphi_{i}^{0}+b_{\varphi_{i}^{Z}}-\sum_{m=i-k+1}^{i}\Delta \varphi_{m}^{0}+\varepsilon_{\Delta \varphi_{m}^{Z}}\\ \; & =\varphi_{i-k}^{0}+b_{\varphi_{i}^{Z}}-\sum_{m=i-k+1}^{i}\varepsilon_{\Delta \varphi_{m}^{Z}}\;\mathrm{with}\;\varphi_{i-k}^{0}=\varphi_{i}^{0}-\sum_{m=i-k+1}^{i}\Delta \varphi_{m}^{0} \end{array}$$

With $\mathrm{Var}(\varphi_{i}^{Z})=0$ and $\mathrm{Var}\left(\varphi_{i-k}^{Z}\right)=\sum_{m=i-k+1}^{i}\mathrm{Var}\left(\varepsilon_{\Delta \varphi_{m}^{Z}}\right)=\sum_{m=i-k+1}^{i}{\sigma_{\Delta \varphi_{m}^{Z}}}^{2}$; thus, it holds that

(31a) $$\varphi_{i}^{Z}∼N\left(\varphi_{i}^{0}+b_{\varphi_{i}^{Z}},0\right)$$

(31b) $$\varphi_{i-k}^{Z}∼N\left(\varphi_{i-k}^{0}+b_{\varphi_{i}^{Z}},\sum_{m=i-k+1}^{i}{\sigma_{\Delta \varphi_{m}^{Z}}}^{2}\right)$$

Based on these considerations, a method can now be presented to determine the error offset $b_{\varphi_{i}^{Z}}$. The approach is based on a weighted comparison of the last orientations from ZUPT $\varphi_{i}^{Z}$ and UWB $\varphi_{i}^{U}$.

The weights are determined from the inverse variance of each directional comparison. Here, the index *k* denotes a stride *k* time steps in the past (see (24)):

(32) $$w_{i-k}=\frac{1}{\mathrm{Var}(\varphi_{i-k}^{Z}-\varphi_{i-k}^{U})}=\frac{1}{\mathrm{Var}\left(\varphi_{i-k}^{Z}\right)+\bar{\mathrm{Var}(\varphi^{U})}}\;\mathrm{with}\;k>0$$

Here, $\bar{\mathrm{Var}(\varphi^{U})}$ is constant and represents the mean expected dispersion of the UWB orientation. In contrast, $\mathrm{Var}(\varphi_{i-k}^{Z})$ increases corresponding to (31b) with each past stride *k*.

In the following, we describe how the last *n* weighted orientations from ZUPT $\varphi_{i}^{Z}$ are rotated to the corresponding orientations of UWB $\varphi_{i}^{U}$ to determine $b_{\varphi_{i}^{Z}}$. The approach is based on the weighted average difference between the two histories. The orientation differences $\Delta \varphi_{i-k}^{ZU}=\varphi_{i-k}^{Z}-\varphi_{i-k}^{U}$ are treated as vectors of orientation $\Delta \varphi_{i-k}^{ZU}$ and magnitude $w_{i-k}$ and summed. The result is a vector with an orientation corresponding to the weighted average orientation difference [47]:

(33) $$\bar{{\Delta \varphi }_{i}^{ZU}}=2\left(\sum_{k=1}^{n}w_{i-k}\cdot \sin{\Delta \varphi }_{i-k}^{ZU},\sum_{k=1}^{n}w_{i-k}\cdot \cos{\Delta \varphi }_{i-k}^{ZU}\right)\approx b_{\varphi_{i}^{Z}}$$

Because the orientation errors of the UWB measurements cancel on average for a sufficiently large history and the constantly shifted orientations from ZUPT are weighted according to their proportional errors, the average weighted difference of the two quantities corresponds to the sought unknown offset $b_{\varphi_{i}^{Z}}$. However, the determination of $b_{\varphi_{i}^{Z}}$, and consequently the error of $\varphi_{i}^{U}$, is an estimate, as a limited number of past of strides is considered. For this reason, a base value of the dispersion $\bar{\mathrm{Var}(\varphi^{U})}$ is introduced, which shall not be undercut. From the Equations (27), (28), and (33) and the base value of the variance, it follows that

(34) $$\mathrm{Var}\left(\varphi_{i}^{U}\right)\approx \max\left[\frac{\pi }{2}\cdot {\left(\varphi_{i}^{U}-\varphi_{i}^{Z}+\bar{{\Delta \varphi }_{i}^{ZU}}\right)}^{2},\bar{\mathrm{Var}(\varphi^{U})}\right]$$

#### 3.2.4. Virtual Stride Vector—Translation

The endpoint of the virtual stride vector $V_{e}^{U}$ from (7b) specifies the UWB translation, i.e., the position of the UWB measurement for the current user stride. The uncertainty of this position is expressed by the covariance matrix $\Sigma_{V_{e}^{U}}$. The variance estimates of the stride length and direction of the virtual stride vector from Section 3.2.2 and Section 3.2.3 map the uncertainty in polar coordinates. In order for these error estimates to be used to estimate the position error in a Cartesian coordinate system, the previous variance estimates must be suitably transformed.

For small errors, the unscented transform (UT) described in [48] is utilized. However, this transform fails for large variances of the orientation φ. In this case, we develop an alternative method in this section for estimating the position error, then introduce a criterion for deciding between the two methods.

The UT uses sigma points defined at specific locations in the original distribution. In the case considered here, these points represent the dispersion in polar coordinates. They are transformed into Cartesian coordinates and then converted back into a distribution.

The starting point for this is the function f(p) used for transforming the erroneous polar coordinates $p={\left(p_{\varrho },p_{\varphi }\right)}^{T}$ into the Cartesian coordinates $k={\left(k_{x},k_{y}\right)}^{T}$:

(35) $$k=\left(\begin{array}{c} k_{x}\\ k_{y} \end{array}\right)=f\left(p\right)=\left(\begin{array}{c} p_{\varrho }\cdot \cos\left(p_{\varphi }\right)\\ p_{\varrho }\cdot \sin\left(p_{\varphi }\right) \end{array}\right)$$

The respective coordinates are treated as stochastic variables with mean values of $\bar{p}=\left({\bar{p}}_{\varrho },{\bar{p}}_{\varphi }\right)$ and $\bar{k}=\left({\bar{k}}_{x},{\bar{k}}_{y}\right)$ and the associated covariance matrices

(36a) $$p∼N(\bar{p},\Sigma_{p})$$

(36b) $$k∼N(\bar{k},\Sigma_{k})$$

with

(37a) $$\Sigma_{p}=\left[\begin{array}{cc} \mathrm{Var}(p_{\varrho }) & \mathrm{Cov}(p_{\varrho },p_{\varphi })\\ \mathrm{Cov}(p_{\varphi },p_{\varrho }) & \mathrm{Var}(p_{\varphi }) \end{array}\right]$$

(37b) $$\Sigma_{k}=\left[\begin{array}{cc} \mathrm{Var}(k_{x}) & \mathrm{Cov}(k_{x},k_{y})\\ \mathrm{Cov}(k_{y},k_{x}) & \mathrm{Var}(k_{y}) \end{array}\right]$$

In the UT, the angular component of the sigma points in polar coordinates $P_{\varphi ,m}$ can adopt values above π/2 when the variance of orientation in the original distribution is large. Thus, the covariance matrix transformed by the trigonometric terms in (35) is increasingly insufficient to model the dispersion caused by the uncertainty of the angular component $p_{\varphi }$. In the extreme case, the sideways scattering is no longer modeled at all. For this reason, the angular component of the *n* sigma points $P_{m}=\left(P_{\varphi ,m},P_{\varrho ,m}\right)$ (with 0<m<n) is reduced to the interval ${\bar{p}}_{\varphi }-\frac{\pi }{2}\leq P_{\varphi ,m}\leq {\bar{p}}_{\varphi }+\frac{\pi }{2}$:

(38) $$P_{\varphi ,m}=\left\{\begin{array}{cc} {\bar{p}}_{\varphi }-\frac{\pi }{2} & \mathrm{for}\;P_{\varphi ,m}\leq {\bar{p}}_{\varphi }-\frac{\pi }{2}\\ {\bar{p}}_{\varphi }+\frac{\pi }{2} & \mathrm{for}\;P_{\varphi ,m}\geq {\bar{p}}_{\varphi }+\frac{\pi }{2}\\ P_{\varphi ,m} & \mathrm{else} \end{array}\right.$$

Only when all sigma points are within this interval is the orientation error considered small enough for UT to be applied:

(39) $$\Sigma_{p}=\mathrm{diag}\left(\mathrm{Var}\left(p_{\varrho }\right),\mathrm{Var}\left(p_{\varphi }\right)\right)=\mathrm{diag}\left(\mathrm{Var}\left(\varrho^{U}\right),\mathrm{Var}\left(\varphi^{U}\right)\right)\overset{\mathrm{UT}}{⟼}\Sigma_{\mathrm{UT}}^{V_{e}^{U}}4$$

Here, $\mathrm{Var}(\varrho^{U})$ is known from (21) and $\mathrm{Var}(\varphi^{U})$ is known from (34). The expected value in Cartesian coordinates $\bar{k}=V_{e}^{U}$ is known from (7b), and the expected value in polar coordinates $\bar{p}=\varphi^{U}$ is known from (5).

For large errors, the Wasserstein-2 distance $W_{2}$, which in the application considered here is known as the Fréchet distance or Earth-Movers Metric [49,50,51,52], is used to model the position error instead. For two normally distributed multidimensional quantities *A* and *B* with means $\mu_{A},\mu_{B}$ and covariance matrices $\Sigma_{A},\Sigma_{B}$, it holds that [51,52]

(40) $$\begin{array}{cc} W_{2} & =\min_{A,B}E{∥A-B∥}_{2}\\ \; & =\sqrt{{∥\mu_{A}-\mu_{B}∥}_{2}^{2}+\mathrm{tr}\left(\Sigma_{A}\right)+\mathrm{tr}\left(\Sigma_{B}\right)-2{\left[\mathrm{tr}\left(\Sigma_{A}\Sigma_{B}\right)+2\sqrt{\det(\Sigma_{A}\Sigma_{B})}\right]}^{1/2}} \end{array}$$

In the case considered here, the expression $\min_{A,B}E{∥A-B∥}_{2}^{2}$ is interpreted as the smallest possible mean Euclidean distance between the realized pairs of values of two random variables. This fact can be applied to the comparison of the erroneous stride vectors from UWB and ZUPT to obtain an estimate for the position error of the UWB measurement. Here, the same origin is assumed for both vectors and the $W_{2}$ distance between the error distributions of the vector endpoints is determined. This metric serves as an estimate of the lower bound for the expected distance between the two position estimates from UWB and ZUPT. This mean distance or MAD is then transformed into a variance estimate, which is ultimately assumed to be isotropic and is used to evaluate the UWB dispersion in Cartesian space. This process is described in detail below.

The error variance of the virtual stride vector from UWB is modeled as a zero matrix, and as such is treated as a point for which the $W_{2}$ distance is to be determined. The reference vector from ZUPT has known variances for the vector length $\mathrm{Var}(\varrho^{Z})$ from (12) and the estimated orientation $\mathrm{Var}\left(\varphi^{Z}\right)=\mathrm{Var}\left(\hat{\varphi }\right)$, which is taken from the state variance estimate of the orientation filter in (50). The error variance of the reference vector in polar coordinates $\Sigma_{p}^{Z}$ is transferred to the Cartesian coordinate system by the UT to obtain $\Sigma_{k}^{Z}$:

(41) $$\begin{array}{cc} \Sigma^{U} & =\mathrm{diag}(0,0)\\ \Sigma_{p}^{Z} & =\mathrm{diag}\left(\mathrm{Var}\left(\varrho^{Z}\right),\mathrm{Var}\left(\varphi^{Z}\right)\right)\overset{\mathrm{UT}}{⟼}\Sigma_{k}^{Z} \end{array}$$

The components of the expected value of the reference vector ${\bar{p}}^{Z}=\left({\bar{p}}_{\varrho },{\bar{p}}_{\varphi }\right)$ are measured directly using ZUPT and taken from (49) as ${\bar{p}}_{\varphi }=\hat{\varphi }$, respectively, and converted by UT to obtain $k^{Z}$. The expected value of the UWB position in Cartesian coordinates ${\bar{k}}^{U}$ is known as $V_{e}^{U}$ from (7b). Then, the $W_{2}$ distance between the two transformed distributions is calculated:

(42) $$\begin{array}{cc} W_{2} & =\min_{k^{U},k^{Z}}E{∥k^{Z}-k^{U}∥}_{2}\\ \; & =\sqrt{{∥{\bar{k}}^{Z}-V_{e}^{U}∥}_{2}^{2}+\mathrm{tr}\left(\Sigma_{k}^{Z}\right)+\mathrm{tr}\left(\Sigma^{U}\right)-2{\left[\mathrm{tr}\left(\Sigma_{k}^{Z}\Sigma^{U}\right)+2\sqrt{\det(\Sigma_{k}^{Z}\Sigma^{U})}\right]}^{1/2}}\\ \; & =\sqrt{{∥{\bar{k}}^{Z}-V_{e}^{U}∥}_{2}^{2}+\mathrm{tr}\left(\Sigma_{k}^{Z}\right)}\;\mathrm{with}\;\Sigma^{U}=\mathrm{diag}\left(0,0\right) \end{array}$$

This distance is interpreted as the MAD between a reference (stride vector from ZUPT) and a measurement (virtual stride vector from UWB), and is transformed into an isotropic variance estimate of the position at the endpoint of the virtual UWB stride vector $V_{e}^{U}$ using (10):

(43) $$\Sigma_{W_{2}}^{V_{e}^{U}}=\left[\begin{array}{cc} \frac{\pi }{2}{\left(W_{2}\right)}^{2} & 0\\ 0 & \frac{\pi }{2}{\left(W_{2}\right)}^{2} \end{array}\right]$$

Due to the isotropy of the covariance matrix, the directional information of small dispersions is lost; thus, this estimate is only used when there are large deviations between the ZUPT reference and the UWB vector. Larger deviations indicate a significant error that may persist across multiple strides. In this case, the formulation just described allows for stronger error correction and more accurate continuation of the user path through PDR/ZUPT.

Finally, for the uncertainty of the UWB position at the top of the virtual stride vector $\Sigma_{V_{e}^{U}}$, it holds that

(44) $$\Sigma_{V_{e}^{U}}=\left\{\begin{array}{cc} \Sigma_{W_{2}}^{V_{e}^{U}} & \mathrm{if}\;\mathrm{UT}\;\mathrm{constrained}\\ \Sigma_{\mathrm{UT}}^{V_{e}^{U}} & \mathrm{else} \end{array}\right.$$

with $\Sigma_{W_{2}}^{V_{e}^{U}}$ from (43) and the possibly restricted covariance $\Sigma_{\mathrm{UT}}^{V_{e}^{U}}$ via UT from (39).

### 3.3. Fusion of ZUPT and Virtual Stride Vector

The presented system consists of two filters: a Kalman filter to determine user orientation, and an Extended Kalman Filter (EKF) to determine user position. Both filters use components of the virtual stride vector and the ZUPT stride vector for state estimation.

#### 3.3.1. Orientation Filter

The orientation filter is implemented as a Kalman filter. The state to be estimated is the orientation ${\hat{\varphi }}_{i}$ at the current time *i*. With each stride, the orientation estimate is updated. The user orientation is estimated as a linear process

(45) $$\varphi_{i}=\varphi_{i-1}+\Delta \varphi_{i}+\varepsilon_{\Delta \varphi ,i}$$

disturbed by an error component $\varepsilon_{\Delta \varphi ,i}$ that is normally distributed around zero and has variable variance $Q_{\Delta \varphi ,i}$

(46) $$\varepsilon_{\Delta \varphi ,i}∼N\left(0,Q_{\Delta \varphi ,i}\right)$$

Here, $Q_{\Delta \varphi ,i}$ is dimensioned according to (14) and the orientation change Δφ measured by ZUPT serves as the control input of the state prediction

(47) $${\varphi_{i}}^{-}=\varphi_{i-1}+\Delta \varphi_{i}$$

with the propagated state variance

(48) $${P_{i}}^{-}=P_{i-1}+Q_{\Delta \varphi ,i}.$$

The orientation measurement from UWB $z_{\varphi ,i}=\varphi_{i}^{U}$ from (5) is applied to the state estimate, and the iteration of the orientation estimate is completed as follows:

(49) $${\hat{\varphi }}_{i}={\varphi_{i}}^{-}+K_{i}\left(z_{\varphi ,i}-{\varphi_{i}}^{-}\right)$$

(50) $$P_{i}=\left(1-K_{i}\right)P_{i}^{-}.$$

Both of the above are obtained using the Kalman gain

(51) $$K_{i}=\frac{P_{i}^{-}}{P_{i}^{-}+R_{i}}$$

using $\mathrm{Var}(\varphi_{i}^{U})$ from (34) as $R_{i}$.

#### 3.3.2. Position Filter

The position filter is implemented as an EKF. This filter estimates the position as a two-dimensional state $x={\left(p_{x},p_{y}\right)}^{T}$. The state prediction of the position is accomplished using the step vector $u={\left(\varrho^{Z},\hat{\varphi }\right)}^{T}$ with the measured step length $\varrho^{Z}$ from ZUPT and the orientation estimate of the orientation filter $\hat{\varphi }$ from (49). The endpoint of the virtual stride vector from (7b) serves as the reference measurement $V_{U}^{e}={\left(z_{x},z_{y}\right)}^{T}$ for the position filter.

The position change is modeled as a nonlinear process

(52) $$\begin{array}{cc} x_{i} & =f(x_{i-1},u_{i},w_{i})\\ \; & =x_{i-1}+\left(\varrho_{i}^{Z}+w_{\varrho^{Z},i}\right)\left(\begin{array}{c} \cos({\hat{\varphi }}_{i}+w_{\hat{\varphi },i})\\ \sin({\hat{\varphi }}_{i}+w_{\hat{\varphi },i}) \end{array}\right) \end{array}$$

using the normally distributed error component $w_{i}$ with variance $Q_{i}$

(53) $$\begin{array}{cc} w_{i} & ∼N(0,Q_{i})\\ \left(\begin{array}{c} w_{\varrho^{Z},i}\\ w_{\hat{\varphi },i} \end{array}\right) & ∼N\left(\left(\begin{array}{c} 0\\ 0 \end{array}\right),\left(\begin{array}{cc} Q_{\varrho^{Z},i} & 0\\ 0 & Q_{\hat{\varphi },i} \end{array}\right)\right) \end{array}$$

The variance of the length estimate $Q_{\varrho^{Z},i}$ corresponds to the error model of the step length determination by ZUPT in (12). The variance of the orientation estimate $Q_{\hat{\varphi },i}$ is taken directly from the estimate of the state variance of the orientation filter in (50).

This yields the formulation for the state prediction of the position filter

(54) $$\begin{array}{cc} {\hat{x}}_{i}^{-} & =f({\hat{x}}_{i-1},u_{i},0)\\ \; & ={\hat{x}}_{i-1}+\varrho_{i}^{Z}\left(\begin{array}{c} \cos\left({\hat{\varphi }}_{i}\right)\;\sin\left({\hat{\varphi }}_{i}\right) \end{array}\right) \end{array}$$

with the corresponding state variance

(55) $${P_{i}}^{-}=P_{i-1}+W_{i}Q_{i}W_{i}^{T}$$

using the Jacobian matrix $W_{i}$ of the partial derivatives of f(…) with respect to *w*.

(56) $$W_{i}=\frac{\delta f}{\delta w}\left({\hat{x}}_{i-1},u_{i},0\right).$$

The end point coordinate of the virtual stride vector $V_{e,i}^{U}=z_{i}$ is applied directly for position prediction:

(57) $${\hat{x}}_{i}={\hat{x}}_{i}^{-}+K_{i}\left(z_{i}-{\hat{x}}_{i}^{-}\right)$$

Finally, the state variance is updated as follows:

(58) $$P_{i}=\left(I_{2\times 2}-K_{i}\right)P_{i}^{-}$$

Both of the above are obtained using the Kalman gain

(59) $$K_{i}=\frac{P_{i}^{-}}{P_{i}^{-}+R_{i}}$$

with $R_{i}=\Sigma_{V_{e}^{U},i}$ from (44).

## 4. Experiment

The fusion of UWB and PDR was evaluated in three different test environments, one featuring a high density of UWB anchor stations and the other two featuring a lower density. During the test runs, the users held a Comnovo UWB transceiver in their hand. The UWB unit delivered the determined position with a frequency of 7.15 Hz at high anchor density and 8.73 Hz at low anchor density.

Additionally, the users were equipped with a Hillcrest FSM-9 IMU with a sampling rate of 125Hz placed on the top of the users foot. The corresponding ZUPT algorithm detected the swing and stance phases of the users’ gait to produce estimates of their stride length and orientation change at the beginning of the stance phase. The ZUPT thresholds were adapted to each user individually. The algorithm is described in detail in [42]. Users were instructed to hold the UWB receiver approximately above the foot with the IMU. The two devices were connected via USB to a Raspberry Pi 3 on which the measurement data were collected.

The different test environments are described in Section 4.1, and the construction of a ground truth along the test tracks is detailed in Section 4.2.

### 4.1. Environments

The experiments conducted to evaluate the presented methods were performed in three different environments. Each environment included different conditions for localization with UWB, characterized by different degrees of shadowing and number as well different arrangements of the UWB anchors. In order to judge the static conditions for UWB localization, we have included a plot of the horizontal dilution of precision (HDOP) for each environment. Please note that the HDOP is defined by the geometric arrangement of UWB anchor stations, not by objects provoking NLOS.

**Figure 2:**
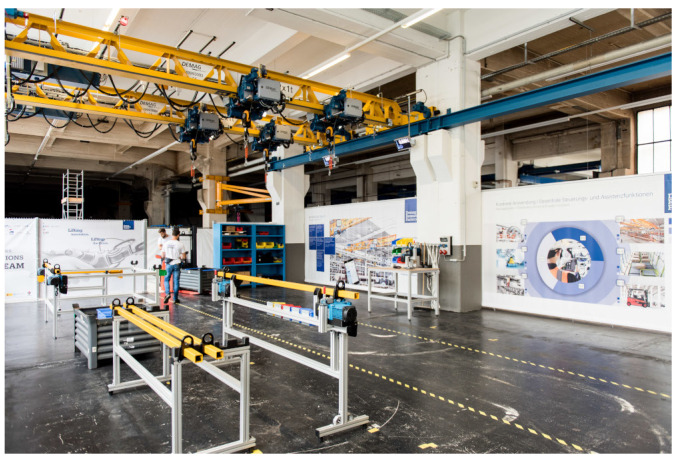
Setup of the Dense environment with high anchor density.

The first environment, hereafter referred to as Dense, was characterized by a high density of UWB anchors. Figure 2 shows the typical setup of this test environment.

The tests were carried out in the Demag Research Factory, which is located in a production hall of Demag Cranes and Components GmbH, located in Wetter an der Ruhr, Germany. For the most part, the mobile UWB transceivers had a clear line of sight to the fixed anchors. During the test runs, however, individual anchors were sporadically obscured by overhead cranes and metallic structures.

**Figure 3:**
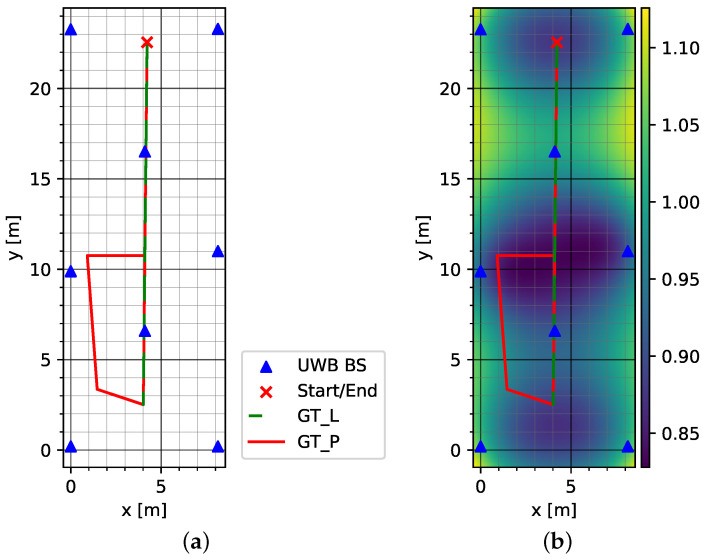
Dense environment with high anchor density: (**a**) test tracks and anchor placement (UWB BS) and (**b**) HDOP. Please note that GT_L is placed on top of a section of GT_P.

Figure 3a shows the test tracks in the environment and the distribution of the UWB anchors (UWB BS). The HDOP plot in Figure 3b consistently shows values of about one.

Two test routes were walked in the test environment Dense. A straight line designated as GT_L led through the center and along the length of the hall. The track was walked along the straight line to the end of the hall and back to the starting point. The second route initially followed the straight line of the first route, changing to a circuit in the left half of the hall and then returning to the straight line in the direction of the starting point. The circuit took place in an area surrounded by metal scaffolding. This route is referred to as GT_P. Each track was walked five times each by three different persons.

**Figure 4:**
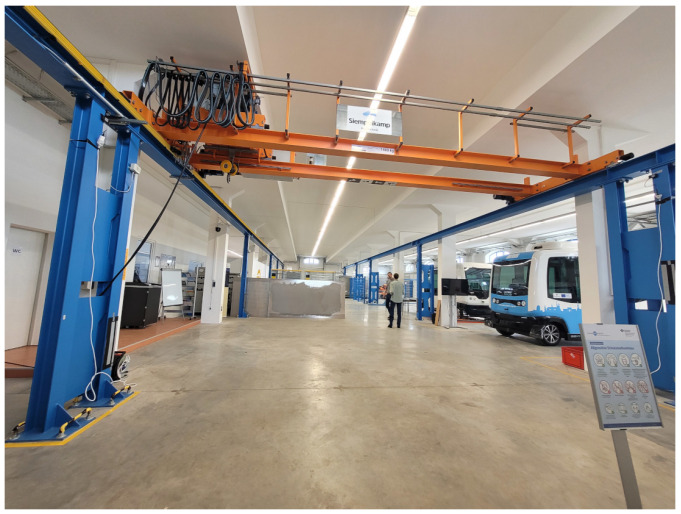
Setup of the Sparse LOS environment with low anchor density.

The second and third environments are referred to as Sparse LOS and Sparse NLOS, respectively. Both environments were set up in Speicher K in the Magdeburg Port of Science, and used the same setup with a limited number of UWB anchors. The environments resembled an industrial hall with an overhead crane, and differed in the routing and placement of metallic objects along the test sections. Figure 4 shows the setup of the test hall in Storage K. In this environment, one of the two test tracks had two start and end points. The test runs started at one point on this track and ended at the other. Both points were used alternately as the respectively start and end points, meaning that half of the tests were walked in opposite directions. Each track was walked four times each by three different persons. The set of participants differed from that in the experiment conducted in the Dense environment. As such, a total of six persons were involved in the test runs.

**Figure 5:**
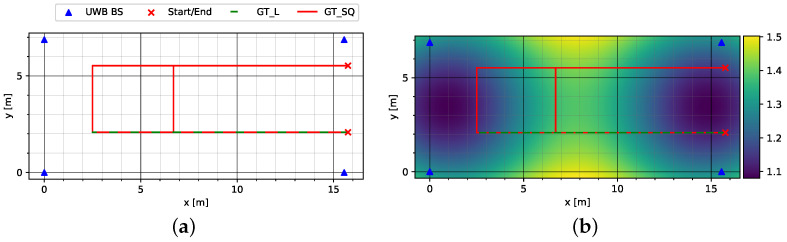
Sparse LOS environment with low anchor density: (**a**) test tracks and anchor placement (UWB BS) and (**b**) HDOP. Please note that GT_L is placed on top of a section of GT_SQ.

In the second environment, Sparse LOS, there were no objects along the tracks, while in the third environment, Sparse NLOS, there were large metallic objects along the track that obscured the line of sight to one or more anchors. Figure 5a shows the routing and placement of the UWB anchors in the Sparse LOS environment. The distribution of HDOP in Figure 5b reveals a maximum with values between 1.4 and 1.5 in the middle of the environment. Here, a test track GT_SQ was followed which connected a straight track with a rectangular circuit, which after a full lap led into a second straight track and terminated at its end. In addition, the initial straight track was walked back and forth as another test track GT_L.

**Figure 6:**
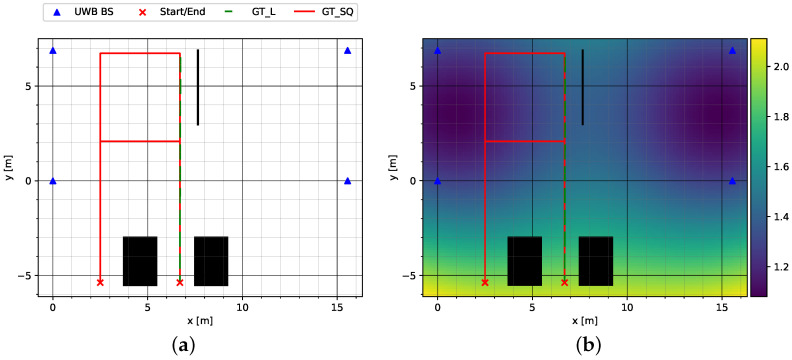
Sparse NLOS environment with low anchor density and shadowing: (**a**) test tracks and anchor placement (UWB BS); (**b**) Sparse NLOS environment with low anchor density and shadowing. Large objects that can lead to NLOS are drawn in black. Please note that GT_L is placed on top of a section of GT_SQ.

In the third and final test environment, Sparse NLOS, which can be seen in Figure 6a, both a round-trip GT_SQ and a straight track GT_L were completed in the same fashion as in Sparse LOS.

**Figure 7:**
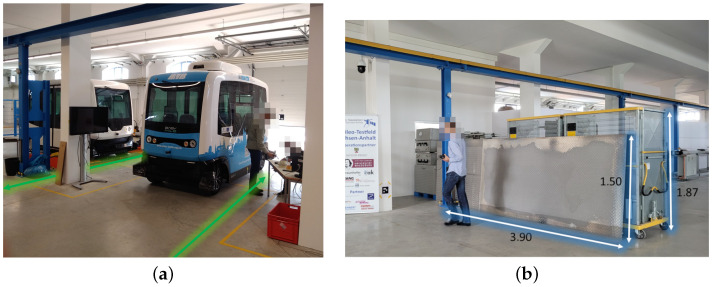
The objects used to obscure the line of sight in the Sparse NLOS environment: (**a**) start and end of test tracks with line of sight strongly obscured by vehicles and concrete pillars; (**b**) large metallic object in the center of the test environment.

However, the track routing was adjusted such that the tracks started outside the area enclosed by the UWB anchors. The HDOP plot in Figure 6b shows that a larger position error is to be expected in these circumstances. Figure 7a shows the environment at the start of the test tracks, which is characterized by shadowing from parked vehicles in addition to the larger HDOP. In addition, a large metallic object was placed along the track obscuring the line of sight from the UWB transceiver in the person’s hand to one or more anchors. The object can be seen in Figure 7b.

### 4.2. Ground Truth

The performed experiments were evaluated using a hybrid ground truth method. The localized persons moved along marked paths, and we derived the position of the person along the length of the path to detect deviations that occurred both parallel and orthogonal to the direction of movement.

The ground truth was marked on the floor of the test environment. The test person attached an IMU to the top of their foot and followed the marked test path as closely as possible with the equipped foot. The resulting representation of the user’s walking motion was then mapped onto the straight sections of the test track. For this purpose, strides with significant directional changes were identified between intervals without significant directional changes (the straight subsections).

**Figure 8:**
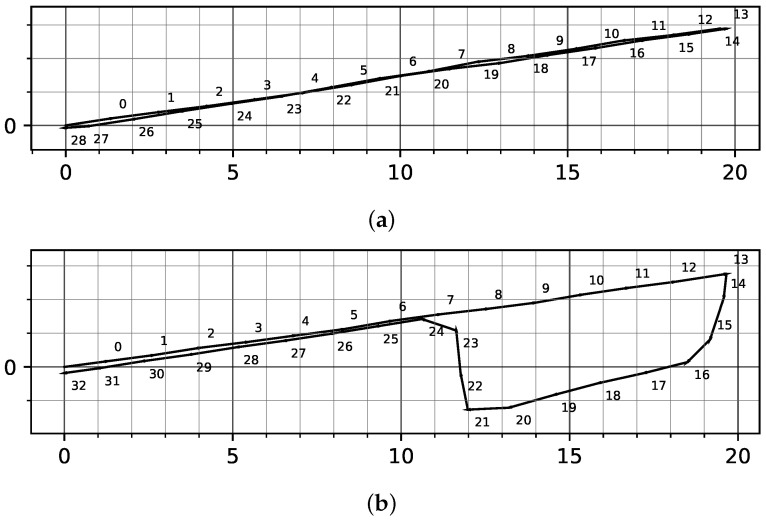
The numbered stride vectors from ZUPT on a straight track and a circuit over several straight subsections: (**a**) straight line to and from, with clear transition between straight sections at stride 13; (**b**) round trip with strides 16 and 24 without clear separation between even subsections. All graph axes are in meters.

Figure 8 shows two exemplary plots of user motion obtained using ZUPT on two different tracks, while Figure 8a shows a straight walking track with the transition between the track subsections clearly separated by two consecutive stride vectors. In Figure 8b, there are strides that lie between the subsections. In the latter case, the subject completed one subsection using the foot without the IMU and continued the movement on the next subsection with the other foot measured here.

**Figure 9:**
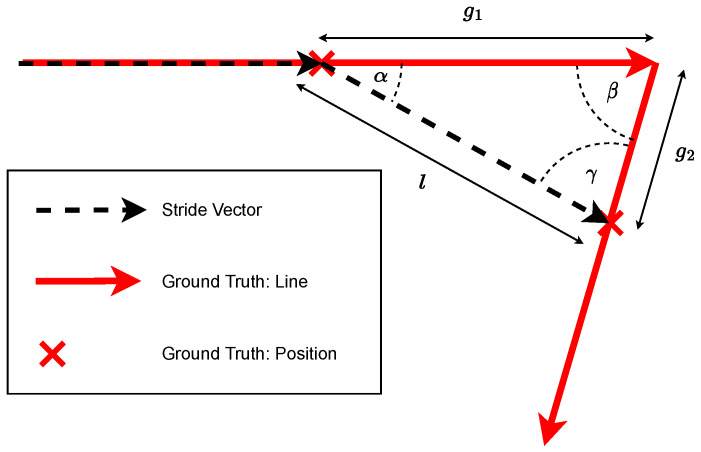
A stride vector between two ground truth subsections.

Thus, because user strides of a single foot may be between subsections, the first stride on a new track section was proportionally divided between the two subsections. Figure 9 shows a stride of length *l* that makes the switch between two ground truth line segments. In order to correctly track the distance traveled on both line segments, it is necessary to consider the remaining track on the original segment $g_{1}$ as well as the track $g_{2}$ traveled with the switching stride on the new segment.

Both partial distances can be calculated by simple trigonometric considerations. A basic assumption here is that the subject is moving parallel to the ground truth before the change. Thus, the change in direction determined by ZUPT can be assumed to be the transition angle α. The angle between the line segments β is known, while the remaining angle γ can be determined by the generally known angle sum in triangles α+β+γ=π. The length of the stride *l* is known from the ZUPT measurement. The two partial distances on the ground truth segments can be determined using the sine theorem:

(60) $$\frac{g_{2}}{\sin\alpha }=\frac{l}{\sin\beta }=\frac{g_{1}}{\sin\gamma }$$

Thus, for the two subsections, it follows that

(61a) $$g_{1}=l\cdot \frac{\sin\gamma }{\sin\beta }$$

(61b) $$g_{2}=l\cdot \frac{\sin\alpha }{\sin\beta }$$

The segments of the user strides in sequence, including the calculated fractions for alternating strides, now map the subject’s locomotion along a straight subsection of known length. The desired actual position along a partial section is derived as follows.

The position $P_{k}$ at the time of stride *k* is modeled as the normalized distance progress along the known length *G* of the straight subsection of the ground truth:

(62) $$P_{k}=\frac{G\cdot \sum_{i=1}^{k}l_{i}}{g_{1}+\sum_{i=1}^{n}l_{i}}\;\mathrm{with}\;l_{1}=g_{2}$$

Here, *n* is the total number of strides on the partial path; in addition, the first user stride $l_{1}$ is described by the proportional locomotion $g_{2}$ from (61b) instead of the measurement from ZUPT. The total distance traveled is extended by the partial distance at segment change $g_{1}$ from (61b). The two partial distances are respectively calculated from different transitions between the subsegments of the ground truth: the transition from the last subsegment to the current one, and the transition from the current segment to the next one.

In a two-dimensional coordinate system with a straight line segment between the start point $G_{s}$ and end point $G_{e}$, it then holds that

(63) $$\begin{array}{cc} P_{k} & =G_{s}+\frac{\left|\vec{G_{e}G_{s}}\right|\cdot \sum_{i=1}^{k}l_{i}}{g_{1}+\sum_{i=1}^{n}l_{i}}\frac{\vec{G_{e}G_{s}}}{\left|\vec{G_{e}G_{s}}\right|}\\ \; & =G_{s}+\frac{\sum_{i=1}^{k}l_{i}}{g_{1}+\sum_{i=1}^{n}l_{i}}\vec{G_{e}G_{s}}\;\mathrm{with}\;l_{1}=g_{2} \end{array}$$

Normalizing the distance progress from ZUPT measurements compensates for errors due to bias in the step length determination. The additive influence of the variance of the length measurement on the variance of the position measurement is compensated for by this at the beginning and end of the subsections. For an average test person, the variance of the position measurement reaches a maximum of 0.002 m^2^ halfway through a 20 m test section. Error influences remain due to user movements that do not precisely follow the given track; while these are partially compensated for by normalization, they cannot be quantified further.

## 5. Results

The utility of fusing stride vectors from ZUPT and position measurements by UWB is evaluated in the following chapter using the positioning error of the tracks and environments described in Section 4.1. In total, eight different modes of fusion are compared, which are detailed in Table 1. The modes differ with respect to the method used to determine the orientation and position measurement variance of the virtual stride vector. In addition to a set of static variances and standard deviations (modes stat_{05, 1, 15, 2, 25}), we evaluated the methods described in Section 3.2 for dynamic estimation of the UWB position error. The dynamic methods differ from each other based on the count of the previous stride vectors used in Section 3.2.3 (modes vec_{5, 10, 15}).

The initial parameters of the fusion are as follows. The initial position and orientation are provided by the first virtual stride vector. The respective initial state uncertainties are parametrized with an orientation standard deviation of $\frac{\pi }{2}$ rad and isotropic position standard deviation of 5 m. The following stride orientation measurement standard deviation is parametrized as $\frac{\pi }{2}$ rad for dynamic modes and as the respective static standard deviation for the static modes. Tn the dynamic modes, the second stride is the initial stride of the stride history, as the preceding virtual stride dictates the initial orientation of the reference ZUPT stride through the initial orientation filter state. Therefore, the first user stride yields no information about the error of the virtual stride. The third and all subsequent strides are processed according to the measurement variances of the respective modes.

The lower bound of orientation standard deviation for the dynamic modes is set to 0.1 rad. The lower bound for the virtual vector length standard deviation is set to 0.12 m. The scaling factor for ZUPT vector length error is set to 0.03 and the angle drift is taken from the FSM-9 datasheet as 0.01 rad/s.

### 5.1. Positioning Accuracy

The results are presented as bar graphs of the mean position error over all test runs on a test track together with the standard deviation. In addition, the tables show the exact values of the statistics. The reference value to the fusion is the accuracy of the positioning by UWB. Here, we distinguish between the error of the last UWB position during a stride (mode uwb) and the endpoint of the virtual stride vector from UWB positions during a stride (mode uwb_vec). The former is shown in the plots, while the latter is used as a measure of position in the fusion evaluated here.

In the diagrams and tables, we ensure that the modes are always compared for the same set of test runs on a single track type. It is possible that for severely perturbed UWB measurements the fused position of certain modes may not converge with the UWB data. These test runs are excluded from the comparisons of all modes in an environment. The fusion is considered convergent if the fused position and the endpoint of the virtual UWB stride vector are no more than 0.5 m apart over three consecutive strides.

#### 5.1.1. Dense LOS Environment

**Figure 10:**
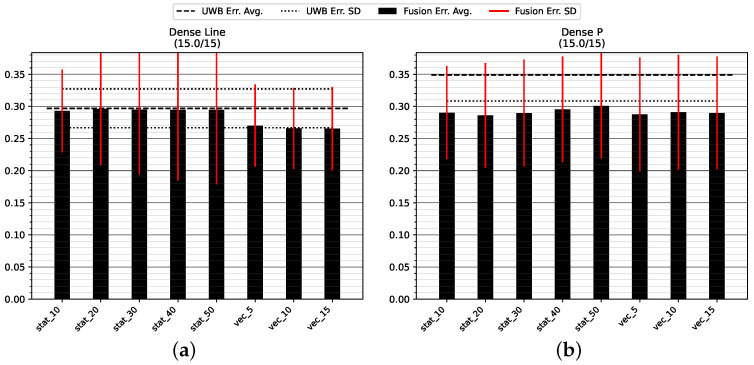
Mean position error and standard deviation in the environment with high anchor density: (**a**) straight track GT_L back and forth (**b**) and roundtrip GT_P. All values are in meters.

Figure 10 and Table 2 show the positioning accuracy in the Dense environment. It can be seen that the fusion approach with dynamic variance estimation results in a better or equivalent positioning compared to an appropriate choice of static fusion. In all cases, the dynamic modes improve the accuracy by one standard deviation of the error when using UWB alone. A closer examination of Table 2 reveals an improvement in accuracy when using the virtual stride vector endpoint (i.e., mode uwb_vec) as a measure of position opposed to the last UWB position measurement (i.e., mode uwb).

**Figure 11:**
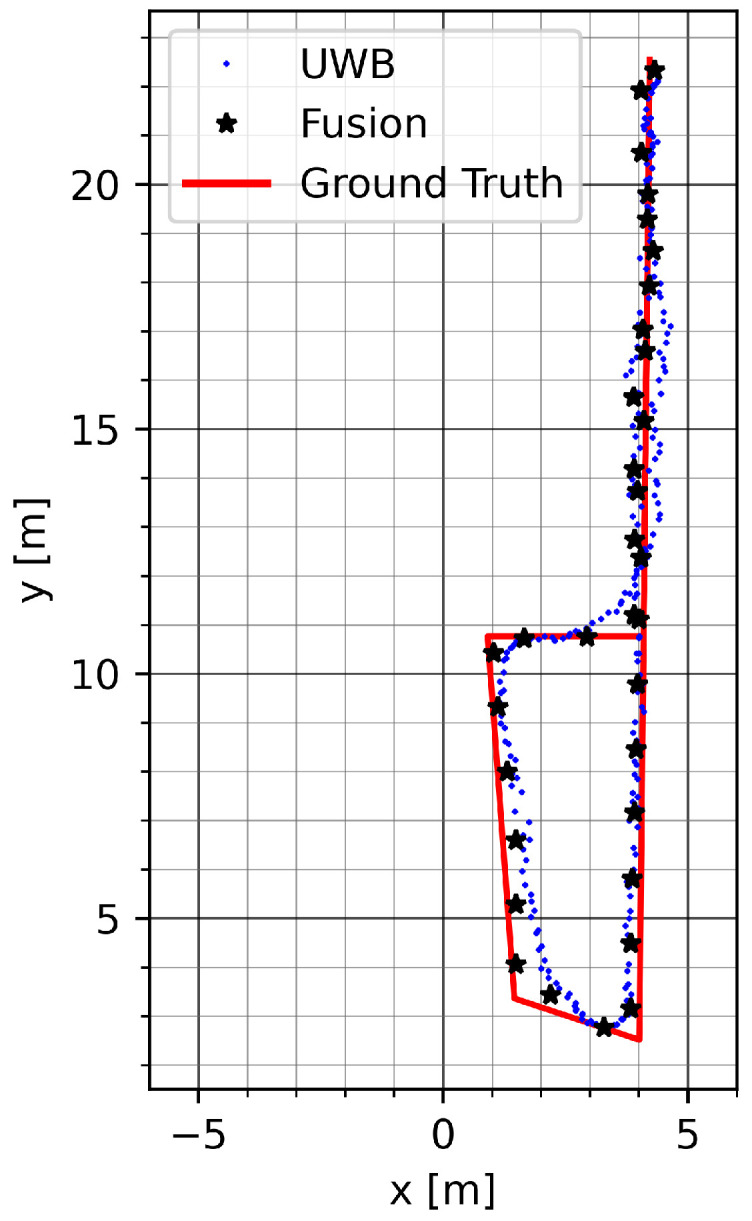
Example run on track GT_P with mode vec_15.

Figure 11 shows a run on track GT_P with mode vec_15. The mitigation of UWB noise can be seen at the beginning on the upper section of the track. The bias is compensated for during the round trip on the bottom of the track.

#### 5.1.2. Sparse LOS Environment

**Figure 12:**
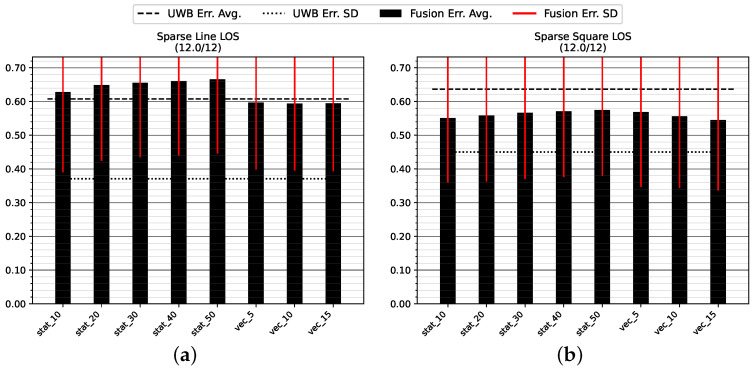
Mean position error and standard deviation in the Sparse LOS test environment with low anchor density: (**a**) straight track GT_L back and forth and (**b**) roundtrip GT_SQ. All values are in meters.

Figure 12 and Table 3 show the positioning accuracy in the Sparse LOS environment. Similar to the results for the Dense environment, the results show improved or equivalent positioning accuracy of the dynamic methods compared to an appropriate choice of static error variance. While the improvement in accuracy through fusion on the GT_L track is negligible, the positioning error of mode uwb_vec is four to five centimeters less than the raw UWB position (mode uwb). Moreover, as seen in Figure 12b and Table 3, the positioning accuracy is improved when using the virtual stride vector endpoint (mode uwb_vec) as a measure of position, and is further improved by fusion with the ZUPT measurement on the longer track GT_SQ.

**Figure 13:**
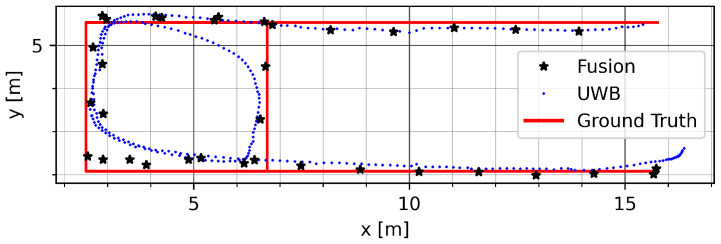
Example run on track GT_SQ in the Sparse LOS environment with mode vec_15.

Figure 13 shows a run on track GT_P with mode vec_15. The distortion of the UWB track during the round trip is largely mitigated by the presented fusion method. A remaining error can be seen on the top part of the track due to an offset in the initial UWB data.

#### 5.1.3. Sparse NLOS Environment

**Figure 14:**
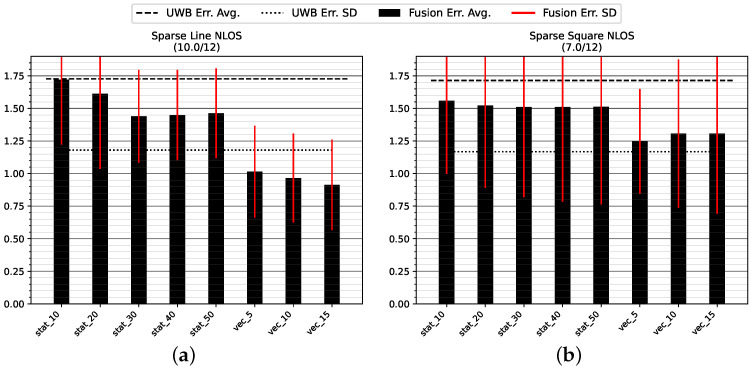
Mean position error and standard deviation in the Sparse NLOS test environment with low anchor density: (**a**) straight track GT_L back and forth (two tracks did not converge); (**b**) roundtrip GT_SQ (five tracks did not converge). All values are in meters.

Figure 14 and Table 4 show the positioning accuracy in the Sparse NLOS environment. Due to large disturbances at the beginning of the test runs, a number of tests did not converge; two tracks were discounted on track GT_L and five tracks failed to converge on track GT_SQ.

The dynamic variance estimation shows a clear advantage over any of the tested static choices for position or orientation measurement variance. There is no discernible trend in the static variance choices that would indicate a choice of higher variance leading to an improved result in this challenging environment. The dynamic modes are superior by about 50 cm of decreased average error on the straight track GT_L and 20 cm on the longer track GT_SQ. There is no clear trend indicating which count of stride history is generally superior.

As with the other experiments, Table 4 indicates that the virtual stride vector endpoint (mode uwb_vec) is a superior measure of position compared to the most recent UWB measurement (mode uwb).

**Figure 15:**
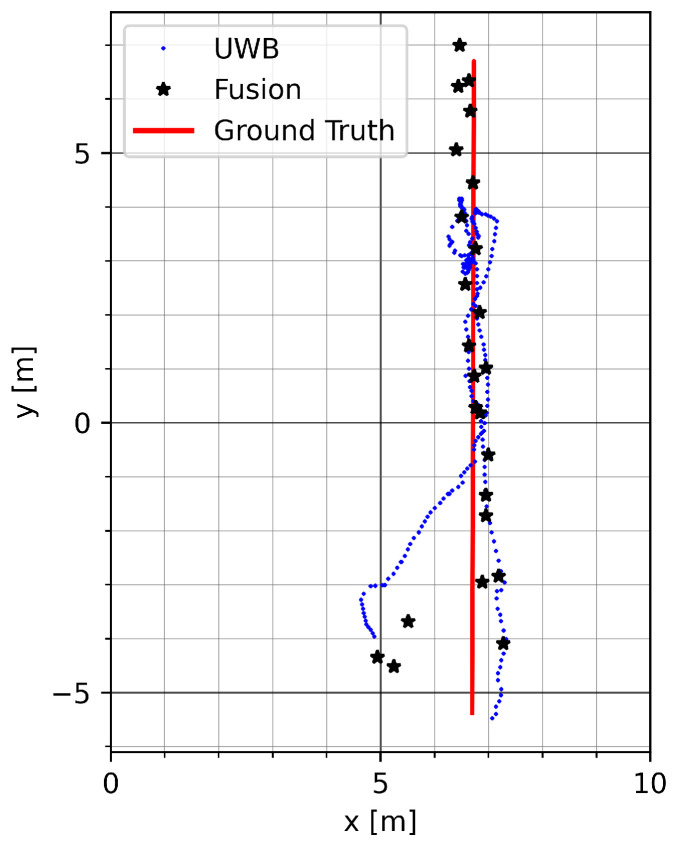
Example run on track GT_L in the Sparse NLOS environment with mode vec_15.

Figure 15 shows a run on track GT_L with mode vec_15. The UWB malfunction on the upper part of the track is mitigated. At the end of the return trip to the bottom, the fused position estimate deviates in the direction of the erroneous UWB measurements due to long-term distortion of UWB.

### 5.2. Attack Compensation

A second experiment was carried out to evaluate the performance in a simulated attack scenario. Coordinated spoofing of UWB positions was assumed, resulting in a deviation of the UWB track from the actual track. In a real-world scenario, this might lead to erroneous movement of a machine following a localized operator, and potentially to subsequent accidents. Therefore, it is of interest to test whether and how the presented method can compensate for these attacks.

The basis for these experiments was the measured data from the previous experiments under LOS. The measured UWB positions were then modified to model the simulated attack. For this, a growing bias vector ${\vec{b}}_{i}$ was introduced at time $T_{m}-T_{b}/2$ that continued to grow until time $T_{m}+T_{b}/2$. Here, the time $T_{m}$ is the midpoint of a test run and $T_{b}$ is the duration of the bias attack. The UWB position $P_{i}$ at time step *i* is modified by the bias ${\vec{b}}_{i}$, resulting in the biased position $P_{b,i}$:

(64) $$P_{b,i}=P_{i}+{\vec{b}}_{i}$$

with the growing bias vector

(65) $${\vec{b}}_{i}={\vec{b}}_{i-1}+{\vec{b}}_{0}*\Delta t$$

where ${\vec{b}}_{0}$ specifies the direction and growth rate of the bias and Δt the time difference between UWB position updates. The simulation was carried out for eight different directions of bias on each track, resulting in the following set of vectors:

(66) $${\vec{b}}_{0}\in \left\{\left(\begin{array}{c} 1\\ 0 \end{array}\right),\left(\begin{array}{c} 1\\ 1 \end{array}\right),\left(\begin{array}{c} 0\\ 1 \end{array}\right),\left(\begin{array}{c} -1\\ 1 \end{array}\right),\left(\begin{array}{c} -1\\ 0 \end{array}\right),\left(\begin{array}{c} -1\\ -1 \end{array}\right),\left(\begin{array}{c} 0\\ -1 \end{array}\right),\left(\begin{array}{c} 1\\ -1 \end{array}\right)\right\}$$

The growth rate is dictated by the vector magnitude $\left|{\vec{b}}_{0}\right|$ and varies between 1 m/s and 1.41 m/s; therefore, it corresponds to the range of a normal adult walking speed [53]. The attack duration $T_{b}$ was chosen to be 10 s.

The resulting positioning accuracy of the disturbed UWB measurements and the fused positions are analyzed in the following sections. The test runs on a track with a certain bias direction are treated as a set of results. For each set, the average positioning error of the modified UWB and fusion is computed. These mean accuracies for a certain bias direction are shown in the graphs as data points for each mode of fusion. A box plot is generated from these data points to judge the dispersion of mean positioning accuracy depending on the bias direction and mode of fusion. The boxes show the 2.5, 25, 50 (median), 75, and 97.5 percentiles, calculated according to Method 8 from [54]. If a test run did not converge for a certain configuration of the bias direction and fusion mode, it was excluded in all other configurations.

#### 5.2.1. Dense LOS Environment

**Figure 16:**
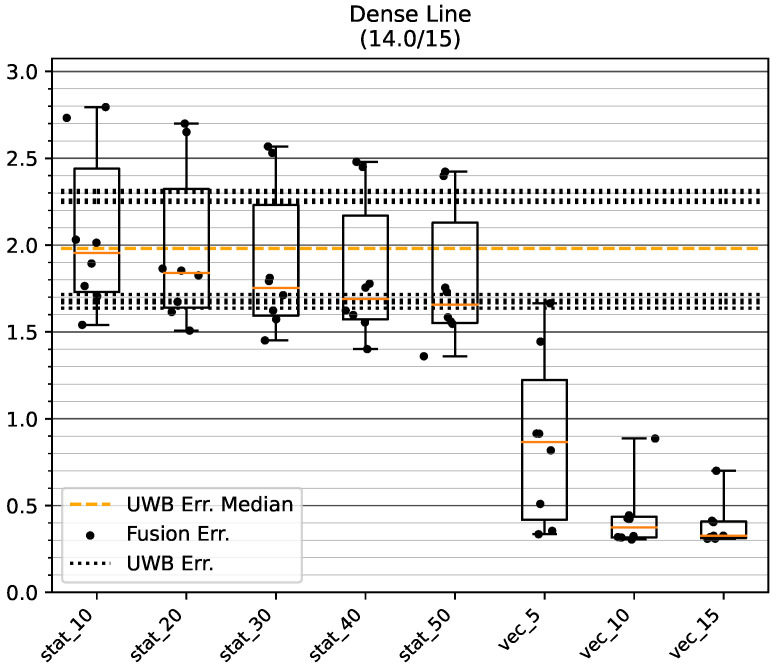
Mean position error of raw UWB and fused position on the GT_L track in the Dense environment. The data points show the mean accuracy for a certain bias direction. All values are in meters. One test run did not converge and was excluded from the analysis.

Figure 16 shows the results of the tests on track GT_L. One track did not converge, and was excluded in all examined configurations to ensure comparability of the results. The dynamic variance estimation shows a median positioning error under 0.5 m for stride histories of length 10 and 15. Indeed, for these modes only one bias direction resulted in an error, of about 0.9 m and 0.7 m, respectively, while all other bias vectors resulted in an error of well under 0.5. Comparing these results with the error range of the raw UWB positions (between about 1.6 m and 2.3 m), a significant improvement in the mean accuracy is apparent when using the presented method. No choice of static UWB measurement variance was able to achieve a consistent improvement in fusion accuracy. For two bias directions, fusion with static variances even resulted in worse mean accuracy compared to the biased UWB data.

**Figure 17:**
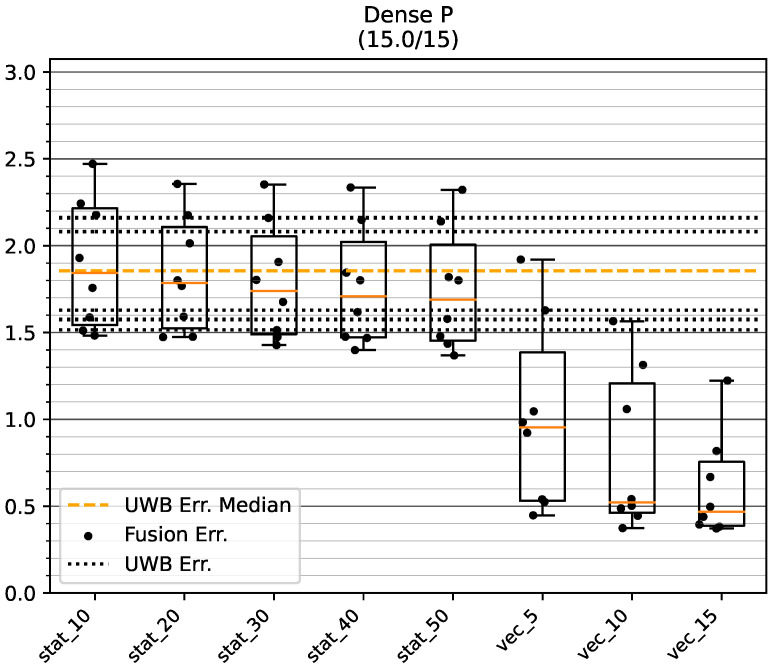
Mean position error of raw UWB and fused position on the GT_P track in the Dense environment. The data points show the mean accuracy for a certain bias direction. All values are in meters.

Figure 17 shows the results of the tests on track GT_P. The performance of the fusion modes is comparable to the tests on the shorter track GT_L. However, a larger dispersion of the mean positioning error for different bias directions can be seen for the dynamic variance estimation. Nonetheless, the median positioning error is situated around 0.5 m for stride histories of length 10 and 15. However, the maximum error increases to about 1.6 m and 1.2 m, respectively.

#### 5.2.2. Sparse LOS Environment

**Figure 18:**
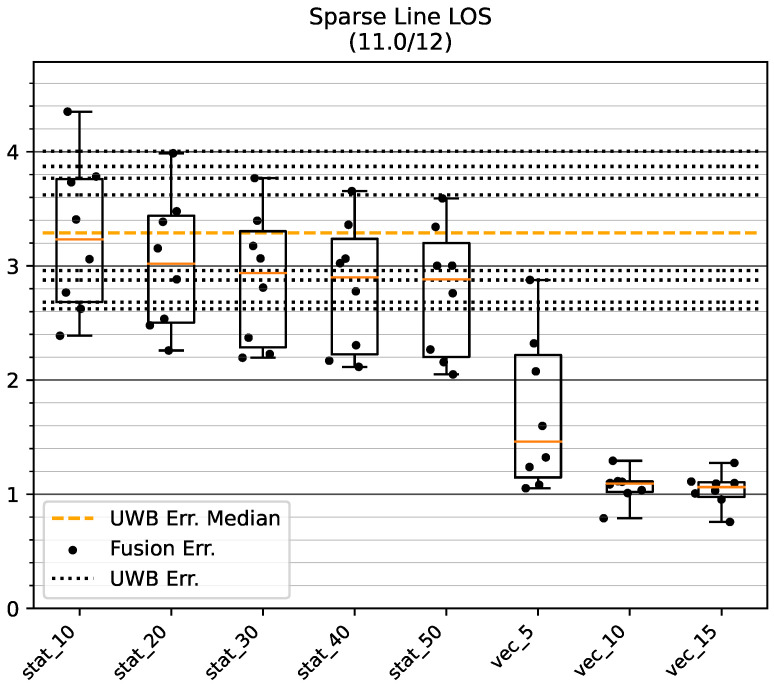
Mean position error of raw UWB and fused position on the GT_L track in the Sparse LOS environment. The data points show the mean accuracy for a certain bias direction. All values are in meters. One test run did not converge and was excluded from the analysis.

The results for the straight track GT_L are shown in Figure 18. One test run failed to converge on this relatively short track and was excluded from all other tests.

Similar to the tests in the Dense environment, the dynamic variance estimation techniques show superior performance compared to any of the static variances, especially for stride histories of length 10 or 15. With a comparatively tight grouping around an error of 1 m, these two dynamic modes are much less affected by different directions of bias compared to the static modes, which exhibit errors in the range from 2 m up to 4.4 m. Dynamic variance estimation with a stride history length of 5 performs better than the static modes, although it shows a large dispersion depending on the direction of bias.

**Figure 19:**
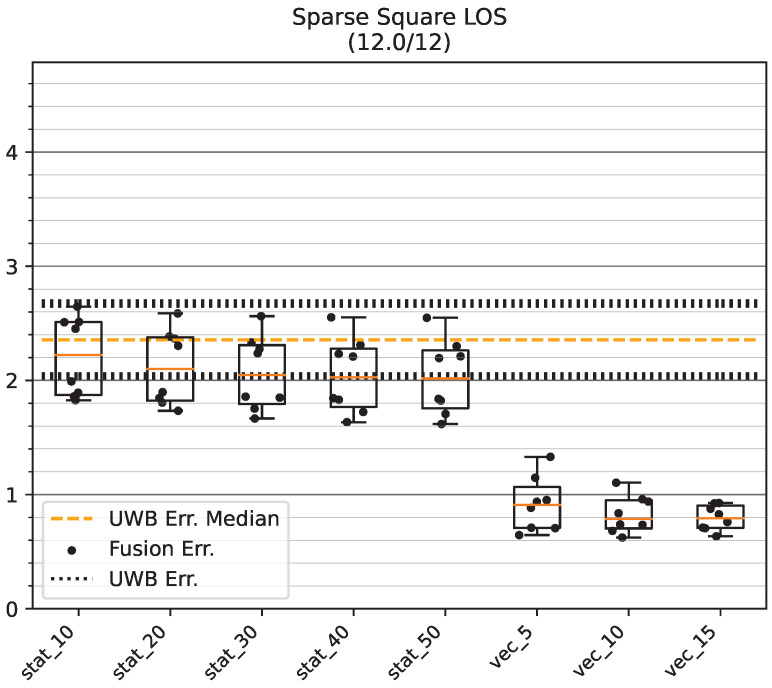
Mean position error of raw UWB and fused position on the GT_SQ track in the Sparse LOS environment. The data points show the mean accuracy for a certain bias direction. All values are in meters.

Figure 19 shows the results on the GT_SQ track. All dynamic variance estimation methods show superior performance, with the remaining mean errors under or around 1 m compared to the mean error of biased UWB (between 2 m and 2.7 m). Static modes show a slight decrease in error that is largely independent of the choice of variance.

### 5.3. Conclusions

The endpoint of the virtual stride vector from UWB measurements is a consistently superior measure of position compared to the last UWB measurement during one stride. Fusion of stride data from ZUPT with this position measure and dynamic variance estimation of that position measurement resulted in improved positioning accuracy on four out of six test tracks and equivalent performance compared to static variances on the remaining two. Overall, the fusion approach resulted in increased accuracy compared to raw UWB data on five out of six test tracks and equivalent performance on the remaining track. The greatest benefit of fusion using dynamic variance estimation is seen in the tests with adverse conditions for UWB. However, the severity of degradation prohibited data fusion in 7 of 24 test runs in the Sparse NLOS environment. It can be seen that the presented method is able to mitigate errors in UWB during several strides; however, the experiments in the sparse NLOS environment show that the presented method is less effective when UWB is degraded severely for longer periods of time.

In the attack scenario, fusion with dynamic variance estimation modes consistently shows better accuracy than fusion with any of the fixed variances. Indeed, the choice of static variance has little and diminishing effect on the mean position accuracy. For the dynamic modes, larger stride histories show a better ability to mitigate positioning error. On the shorter straight tracks, one test run failed to converge. This was likely because of an erroneous initial UWB measurement that was subsequently used to propagate the fused position with ZUPT, thereby propagating the initial error. On longer tracks this error is compensated by following UWB measurements. However, due to the limited track length, the number of compensating UWB measurements was too small prior to error injection by our simulated attack.

**Figure 20:**
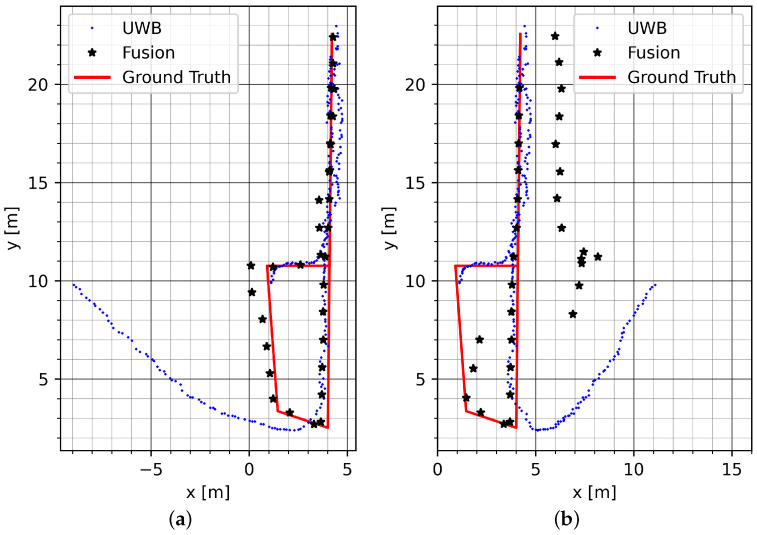
The GT_P track with two directions of simulated bias: (**a**) bias vector ${\left(-1,0\right)}^{T}$ and (**b**) bias vector ${\left(1,0\right)}^{T}$.

Figure 20 shows the same run on track GT_P with two opposing directions of simulated bias. While the introduced error is mitigated for the whole track in Figure 20a, the mitigation in Figure 20b fails after about five strides. This confirms the behavior seen in the Sparse NLOS environment. Severe UWB errors are fully mitigated for several strides; however, if an unfavorable constellation of UWB error and user movement continues for a longer time, the presented method for error mitigation cannot continue to provide reliable user positioning.

## 6. Discussion

In this paper, we have presented a scheme to localize people equipped with UWB transceivers and a shoe-mounted IMU in indoor environments. The proposed approach is intended to improve worker safety and efficiency by detecting, quantifying, and mitigating erroneous UWB measurements.

Our contribution in this work is twofold. First, we show that the endpoint of a virtual stride vector constructed from UWB position measurements during a user stride is a superior measure of user position compared to the last UWB position measurement of that stride. Second, we present a method to compare these virtual stride vectors with stride vectors from ZUPT measurements to derive an error estimate of this position measure. This error estimate is then used to scale the measurement covariance in a fusion scheme to combine the UWB and ZUPT measurements for user localization. This scaling operation is independent of assumptions about the environment or tunable parameters, with the exception of the minimum variances used to prevent overconfidence in the derived virtual vector and dynamic variance estimates. The variance estimates are derived for each stride, and as such are able to respond to sudden increases in error.

We evaluated this fusion scheme in three different industrial environments, finding that it is superior to UWB alone as well as to fusion with fixed variances in three out of four tests during normal operation, and is equivalent in the remaining test. The greatest benefit of our method is seen in an environment with erroneous UWB measurements due to NLOS and unfavorable HDOP. We evaluated our method further by simulating a spoofing attack on the UWB measurements. Here, an even greater benefit is seen in regard to error mitigation. All these experiments were carried out with the same choice of minimum variance, showing that parameter tuning for different situations is not required.

However, while our method can mitigate the positioning error, the range of remaining error seems to depend on the test track type and the direction of injected bias. Further research is needed to draw conclusions about whether the presented method can mitigate all types of position manipulation, and what factors affect the accuracy of the presented method. We suspect that one source of uncertainty is the quality of the initial UWB samples. In our experiments, it is apparent that a large set of past ZUPT vectors is beneficial for error mitigation. The error of the initial UWB measurements is difficult to quantify because of a limited set of past vectors. As such, early biases in UWB that are not properly quantified are not fully mitigated, and can propagate in the filtered position and orientation estimates for a considerable time. In this context, it is necessary to investigate whether localization can continue after large continuing deviations between UWB and ZUPT are detected or whether there needs to be a reset procedure. As the presented error estimation depends on the comparison of past strides, if the past virtual stride vectors show a large systematic bias then the error estimate of the current stride is affected.

In our study, this method was demonstrated using an IMU on the user’s shoe. However, it is conceivable to use a wearable IMU that derives stride length and orientation from other body parts, e.g., a wristband or belt. Heuristics to derive the stride length and orientation can be trained using the virtual stride vectors from UWB as a reference during normal operation.

## Figures and Tables

**Table 1:** The evaluated modes of the measurement update variance in the orientation and position filters.

Mode	Description
stat_10	Static standard deviation of 0.05 rad & 0.1 m
stat_20	Static standard deviation of 0.1 rad & 0.2 m
stat_30	Static standard deviation of 0.15 rad & 0.3 m
stat_40	Static standard deviation of 0.2 rad & 0.4 m
stat_50	Static standard deviation of 0.25 rad & 0.5 m
vec_5	Dynamic variance by comparison of the last 5 strides
vec_10	Dynamic variance by comparison of the last 10 strides
vec_15	Dynamic variance by comparison of the last 15 strides

**Table 2:** Mean position error (Avg), standard deviation (SD) and the percentage change of mean error compared to the UWB measurement (Avg vs. UWB) in the Dense environment. All values in meters.

Mode	Avg (SD)	Avg vs. UWB
(**a**) Straight track GT_L back and forth		
uwb	0.2969 (0.0302)	0.0000
uwb_vec	0.2837 (0.0313)	−0.0444
stat_10	0.2931 (0.0645)	−0.0128
stat_20	0.2961 (0.0872)	−0.0028
stat_30	0.2950 (0.1010)	−0.0062
stat_40	0.2944 (0.1099)	−0.0084
stat_50	0.2946 (0.1160)	−0.0077
vec_5	0.2702 (0.0638)	−0.0898
vec_10	0.2656 (0.0633)	−0.1053
vec_15	0.2654 (0.0650)	−0.1060
(**b**) Roundtrip GT_P		
uwb	0.3489 (0.0405)	0.0000
uwb_vec	0.3320 (0.0463)	−0.0482
stat_10	0.2904 (0.0725)	−0.1676
stat_20	0.2861 (0.0816)	−0.1801
stat_30	0.2897 (0.0832)	−0.1697
stat_40	0.2953 (0.0824)	−0.1535
stat_50	0.3008 (0.0817)	−0.1377
vec_5	0.2876 (0.0885)	−0.1757
vec_10	0.2910 (0.0893)	−0.1659
vec_15	0.2897 (0.0880)	−0.1695

**Table 3:** Mean position error (Avg), standard deviation (SD) and the percentage change of mean error compared to the UWB measurement (Avg vs. UWB) in the Sparse LOS environment. All values in meters.

Mode	Avg (SD)	Avg vs UWB
(**a**) Straight track GT_L back and forth		
uwb	0.6078 (0.2370)	0.0000
uwb_vec	0.5592 (0.2187)	−0.0800
stat_10	0.6283 (0.2378)	0.0337
stat_20	0.6487 (0.2242)	0.0674
stat_30	0.6553 (0.2204)	0.0782
stat_40	0.6606 (0.2198)	0.0868
stat_50	0.6659 (0.2200)	0.0956
vec_5	0.5966 (0.1983)	−0.0184
vec_10	0.5940 (0.1989)	−0.0227
vec_15	0.5948 (0.2014)	−0.0213
(**b**) Roundtrip GT_SQ		
uwb	0.6364 (0.1861)	0.0000
uwb_vec	0.5916 (0.1787)	−0.0704
stat_10	0.5510 (0.1915)	−0.1343
stat_20	0.5585 (0.1954)	−0.1225
stat_30	0.5664 (0.1956)	−0.1100
stat_40	0.5712 (0.1948)	−0.1025
stat_50	0.5747 (0.1940)	−0.0970
vec_5	0.5684 (0.2214)	−0.1069
vec_10	0.5561 (0.2126)	−0.1263
vec_15	0.5451 (0.2091)	−0.1435

**Table 4:** Mean position error (Avg), standard deviation (SD) and the percentage change of mean error compared to the UWB measurement (Avg vs. UWB) in the Sparse NLOS environment. All values in meters.

Mode	Avg (SD)	Avg vs. UWB
(**a**) Straight track GT_L back and forth		
uwb	1.7266 (0.5467)	0.0000
uwb_vec	1.7030 (0.5455)	−0.0137
stat_10	1.7208 (0.4982)	−0.0034
stat_20	1.6118 (0.5765)	−0.0665
stat_30	1.4387 (0.3568)	−0.1667
stat_40	1.4485 (0.3460)	−0.1611
stat_50	1.4630 (0.3435)	−0.1527
vec_5	1.0145 (0.3528)	−0.4125
vec_10	0.9659 (0.3408)	−0.4406
vec_15	0.9127 (0.3477)	−0.4714
(**b**) Roundtrip GT_SQ		
uwb	1.7146 (0.5467)	0.0000
uwb_vec	1.6733 (0.5418)	−0.0241
stat_10	1.5586 (0.5611)	−0.0910
stat_20	1.5223 (0.6321)	−0.1121
stat_30	1.5101 (0.6916)	−0.1192
stat_40	1.5109 (0.7275)	−0.1188
stat_50	1.5129 (0.7499)	−0.1176
vec_5	1.2473 (0.4008)	−0.2725
vec_10	1.3066 (0.5701)	−0.2380
vec_15	1.3066 (0.6139)	−0.2380

## Data Availability

The data presented in this study are available on request from the corresponding author. The data are not publicly available due to pending discussions with involved parties.

## Abbreviations

The following abbreviations are used in this manuscript:

EKF	Extended Kalman Filter
HDOP	Horizontal Dilution of Precision
IMU	Inertial Measurement Unit
KF	Kalman Filter
LIDAR	Light Detection and Ranging
LOS	Line Of Sight
MAD	Mean Absolute Deviation
NLOS	Non-Line of Sight
PDR	Pedestrian Dead Reckoning
UT	Unscented Transform
UWB	Ultra-Wideband
ZUPT	Zero Velocity Update
